# From Pharmacovigilance Signals to Mechanistic Phenotypes: Integrating ADMET, PK/PD, and Network Context to Interpret Antiviral Safety in Pregnancy

**DOI:** 10.3390/ph19030450

**Published:** 2026-03-11

**Authors:** Bárbara Costa, Nuno Vale

**Affiliations:** 1 PerMed Research Group, RISE-Health, Faculty of Medicine, University of Porto, Alameda Professor Hernâni Monteiro, 4200-319 Porto, Portugal; bcosta@med.up.pt; 2 RISE-Health, Department of Community Medicine, Health Information and Decision (MEDCIDS), Faculty of Medicine, University of Porto, Rua Doutor Plácido da Costa, 4200-450 Porto, Portugal; 3 Laboratory of Personalized Medicine, Department of Community Medicine, Health Information and Decision (MEDCIDS), Faculty of Medicine, University of Porto, Rua Doutor Plácido da Costa, 4200-450 Porto, Portugal

**Keywords:** pregnancy pharmacovigilance, antiviral safety, adverse drug reaction phenotypes, network pharmacology, polypharmacy, ADMET prediction, pharmacokinetics in pregnancy

## Abstract

**Background:** Antiviral therapies are widely used during pregnancy and are generally considered safe, pregnancy-specific severe safety signals continue to be observed in post-marketing pharmacovigilance data. These signals are rarely interpreted within an integrated mechanistic framework. **Methods:** We analysed pregnancy-related EudraVigilance reports (2015–2025) using a previously network-based pharmacovigilance framework. Established ADR clusters were treated as fixed phenotypes and integrated with in silico ADMET liabilities, literature-derived pregnancy pharmacokinetic/pharmacodynamic (PK/PD) parameters, polypharmacy and co-medication network metrics, and exploratory statistical, machine-learning, and exposure–liability analyses for mechanistic prioritisation. **Results:** Phenotype membership explained 22.3% of the variance in composite ADMET risk (intraclass correlation coefficient = 0.223; *p* < 0.001), and all tested ADMET parameters differed significantly across phenotypes (FDR-adjusted *p* < 10^−10^). One phenotype showed pronounced enrichment, with 13 antivirals over-represented. Polypharmacy strongly modified seriousness, with odds of serious outcomes increasing by ~5% per additional co-reported active drug (OR 1.05, 95% CI 1.04–1.05). A composite mechanistic vulnerability index showed moderate concordance with empirical burden (Spearman’s ρ = 0.65), while regimen-level prioritisation of drug–drug interactions (DDIs) identified no high-priority combinations. **Conclusions:** Pregnancy-related antiviral ADRs cluster into reproducible phenotypes driven by mechanistic liability and system-level complexity, supporting mechanistically informed prioritisation and targeted pharmacometric follow-up.

## 1. Introduction

Pregnant individuals remain systematically underrepresented in clinical trials, resulting in persistent gaps in evidence regarding the safety of many therapeutics during pregnancy [1]. This limitation is particularly consequential for antiviral drugs, which are widely used for the treatment and prevention of chronic and acute viral infections, including HIV, hepatitis, influenza, and emerging viral diseases [2]. Pregnancy induces profound physiological changes that can alter drug absorption, distribution, metabolism, and elimination, as well as pharmacodynamic sensitivity [3]. As a result, antivirals deemed safe based on non-pregnant adult data or small pregnancy cohorts may display altered exposure profiles and residual adverse drug reaction signals when used in routine clinical care, which in turn complicates the introduction and implementation of new treatment strategies [4,5].

Pharmacovigilance databases such as EudraVigilance offer a unique window onto pregnancy-related drug safety in real-world settings, capturing heterogeneous regimens, comorbidities, and polypharmacy that are difficult to reproduce in trials [6,7,8]. Traditional signal-detection approaches in these systems, based on pairwise disproportionality for single drug–event combinations, are highly effective for first-line signal generation but remain essentially event-centric and only indirectly address regimen complexity, co-reported reactions, or mechanistic context [9,10]. Building on these foundations, network- and cluster-based analyses have emerged as complementary tools that model adverse drug reactions (ADRs) as structured patterns of co-occurrence, enabling the identification of syndromic safety profiles and regimen-level effects that are not visible to pairwise methods alone [11,12,13]. Beyond traditional pairwise disproportionality, several groups have demonstrated that pharmacovigilance data can be quantitatively linked to drug targets, receptor-binding profiles, and pharmacokinetic/pharmacodynamic (PK/PD) properties to generate mechanistic hypotheses about observed ADR patterns [14,15,16]. Pharmacovigilance–pharmacodynamic and pharmacovigilance–PK/PD frameworks have, for example, correlated FDA Adverse Event Reporting System (FAERS)-derived disproportionality estimates with receptor affinity or transporter inhibition metrics to explain antidepressant-induced hyponatraemia [14] and serotonin syndrome with linezolid–serotonergic drug combinations [17]. Parallel efforts have assembled integrated Absorption, Distribution, Metabolism, Excretion, and Toxicity (ADMET)–ADR resources and (Artificial Intelligence/Machine Learning) AI/ML-driven toxicity models, showing that large curated drug–property–ADR datasets can support predictive modelling of mechanistic liabilities and help rationalise late-stage safety failures [18,19]. Although a few algorithms are beginning to incorporate more complex outcomes, current implementations remain largely confined to general adult populations and still focus predominantly on single ADRs or organ-specific outcomes rather than on structured, syndromic phenotypes under conditions of substantial polypharmacy and altered physiology [6,20].

Building on this trend, we recently applied a network- and cluster-based pharmacovigilance framework to pregnancy-associated antiviral reports in EudraVigilance, showing that co-reported reactions aggregate into stable, reproducible ADR phenotypes that behave as pregnancy-specific safety profiles rather than isolated events [21]. In the present study, we extend a previously developed network- and cluster-based pharmacovigilance framework for pregnancy-related antiviral safety by adding mechanistic and pharmacometrics dimensions. We treat established ADR clusters as stable phenotypic safety profiles and annotate them with in silico ADMET predictors (transporter, enzyme, and toxicity-related liabilities), polypharmacy and co-medication network metrics (regimen complexity and system-level context), and curated pregnancy PK/PD information, including trimester-specific exposure ranges and virologic endpoints where available. This integrated framework enables mechanistic interpretation of real-world safety phenotypes beyond disproportionality alone. Therefore, our contribution is threefold: we map mechanistically annotated antiviral ADR phenotypes in pregnancy, propose drug- and regimen-level vulnerability scores, and quantify exposure–liability relationships under pregnancy-relevant PK bounds to identify antivirals warranting targeted follow-up.

## 2. Results

### 2.1. Composition, Coverage, and Complexity of the Mechanistic Integration

All analyses were anchored in the pregnancy Individual Case Safety Report (ICSR) dataset (N = 1938 unique cases), which formed the sole analytical population throughout. Regression models, seriousness summaries, phenotype mapping, and drug-level aggregations were all derived from this pregnancy-only dataset. For mechanistic integration and reaction-level analyses, case records were structurally expanded to retain each MedDRA Preferred Term and drug–ADR linkage, generating 17,058 reaction-level records. This expansion involved reshaping the 1938 cases to support phenotype clustering, frequency mapping, and mechanistic enrichment; no additional cases were introduced. Case-level analyses were performed on the deduplicated dataset to account for non-independence. Supplementary Table S1 provides the units of analysis and denominators for the analytical components.

Across the 1938 unique ICSRs, 81.8% met regulatory seriousness criteria, with no missing data for seriousness classification. Reports were mapped to five harmonised ADR phenotypes and 25 primary active drugs (Table 1). Polypharmacy burden was substantial, with a median of 11 active drugs per report (IQR 6–27), reflecting extensive concomitant medication exposure in this cohort. Combination therapy predominated—89.1% of reports involved three or more active drugs, and pharmacokinetic boosters were present in 23.6% of ICSRs; no monotherapy cases were reported.

The co-medication network showed high connectivity, with a median mean degree of 43.8 (IQR 40.6–52.4), indicating dense drug co-occurrence patterns across reports. In case-level logistic regression, each additional active drug was associated with a 3% increase in the odds of a serious outcome (OR 1.03, 95% CI 1.02–1.05). This association remained stable when restricting to female, non-follow-up, non-literature reports (OR 1.04, 95% CI 1.02–1.07). Given the high baseline prevalence of seriousness, these odds ratios should be interpreted as incremental associations within an already high-seriousness reporting environment rather than absolute risk differences.

ADMET parameters were available for 22 of 25 drugs included in drug-level modelling. Pregnancy PK metrics were retrieved for 15 of these drugs, and virologic suppression endpoints for 6. Because data completeness varied across analytical domains, each integration component was performed on available-case datasets specific to its analytical level.

### 2.2. Harmonisation of ADR Phenotypes Yields Five Stable Pregnancy Safety Profiles

**Figure 1:**
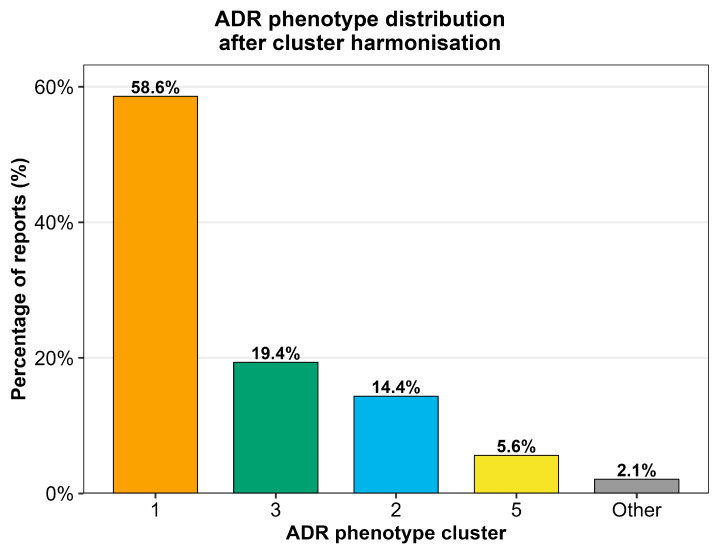
ADR phenotype distribution after cluster harmonisation.

Harmonisation of the previously derived ADR phenotype structure yielded five analytically stable phenotypes (Clusters 1, 2, 3, 5, and Other) by collapsing low-frequency clusters (<2% of reports) into “Other” (Supplementary Table S2). Cluster 1 accounted for the majority of reports, followed by Clusters 3, 2, and 5, with a low-frequency “Other” phenotype (Figure 1). Serious outcome proportions were calculated for each harmonised ADR phenotype (Supplementary Table S3).

### 2.3. ADR Phenotypes Exhibit Marked Differences in Seriousness, Polypharmacy Burden, and Mechanistic Signatures

**Figure 2:**
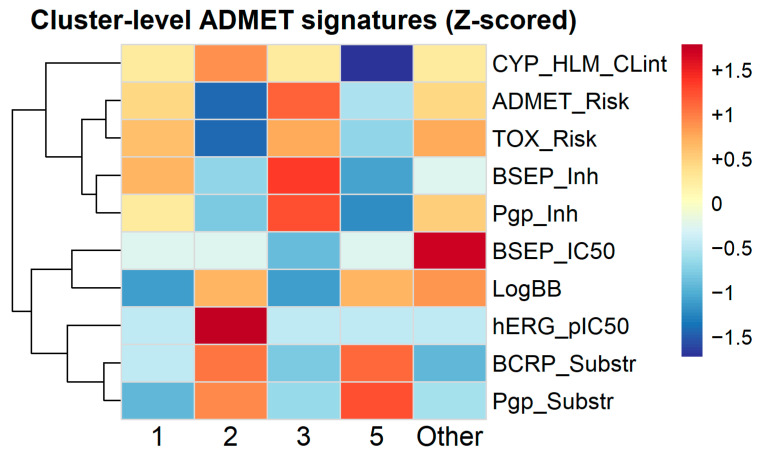
Cluster-level ADMET feature profiles (z-scored). Heatmap of phenotype-level medians (z-scored by variable) for representative ADMET features and liability flags, highlighting differential mechanistic signatures across harmonised phenotypes.

ADR phenotype-level ADMET signatures demonstrated marked heterogeneity across harmonised clusters (Figure 2). Cluster 1 was characterised by elevated composite ADMET_Risk (z = +0.73) and TOX_Risk (z = +0.73), accompanied by comparatively lower hepatic intrinsic clearance (CYP_HLM_CLint; z = −0.73) and hERG liability (z = −0.73). In contrast, Cluster 2 exhibited reduced composite risk (ADMET_Risk z = −1.10; TOX_Risk z = −1.10) but increased hepatic clearance (CYP_HLM_CLint z = +1.10) and hERG signal (z = +1.10). Cluster 3 showed pronounced transporter-related deviations, including elevated BSEP inhibition (z = +1.16) and P-glycoprotein inhibition (z = +0.97), alongside reduced P-gp substrate probability (z = −1.35). Cluster 5 demonstrated increased P-gp substrate probability (z = +1.20) and Breast Cancer Resistance Protein (BCRP) substrate signal (z = +1.17) with concurrent reductions in transporter inhibition metrics. The low-frequency “Other” phenotype exhibited markedly elevated blood–brain barrier penetration (LogBB; z = +1.79) and higher Bile Salt Export Pump (BSEP) IC_50_ values (z = +1.79), distinguishing it from the four principal clusters.

Across numeric ADMET variables (ADMET_Risk, TOX_Risk, CYP_HLM_CLint, BSEP_IC50, hERG_pIC50, LogBB), global differences between phenotypes were statistically significant after Benjamini–Hochberg correction (Kruskal–Wallis, adjusted *p* ≤ 8.9 × 10^−10^; several below machine precision), supporting the stability of the mechanistic contrasts observed in Figure 2 (Supplementary Table S4).

Serious outcome proportions differed across phenotypes at the deduplicated ICSR level. Cluster 1 exhibited the highest seriousness among high-volume phenotypes (98.1%), followed by Cluster 3 (77.0%), Cluster 2 (87.4%), and Other (63.6%) (Supplementary Table S3). Cluster 5 demonstrated 100% seriousness; however, this phenotype comprised a single ICSR (N = 1) and therefore does not represent a stable or generalisable estimate.

### 2.4. Distinct Drug–Phenotype Enrichment Patterns Highlight Cluster-Specific Safety Associations

**Figure 3:**
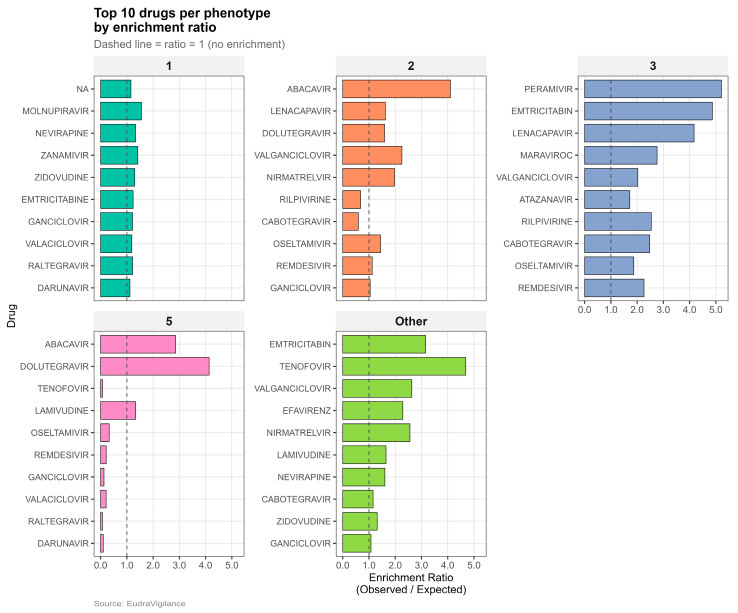
Top enriched drugs per ADR phenotype (observed/expected enrichment ratios).

Across all phenotypes, the top enriched drugs by observed-to-expected enrichment ratio are shown in Figure 3. A summary of the drug–phenotype enrichment matrix is provided in Supplementary Table S5. Contextual disproportionality analyses identified 112 drug–phenotype associations that met multiple-testing correction (FDR q < 0.05), of which the subset with ROR > 1 was considered enriched for mechanistic interpretation. Cluster 3 exhibited the strongest enrichment profile (13 significantly enriched drugs; FDR q < 0.05). Within Cluster 3, the highest RORs were observed for lenacapavir (ROR 14.2, 10 total reports), followed by maraviroc, rilpivirine, cabotegravir, and remdesivir, all with strong statistical support despite relatively modest report counts. In contrast, more widely used agents, such as atazanavir, efavirenz, and darunavir, showed more moderate enrichments (ROR 2.12, 1.71, and 1.54, respectively). They were supported by substantially larger numbers of reports (≥800 total reports per drug), consistent with their long-standing use and broader exposure base. This pattern indicates that Cluster 3 enrichment is shared across both newer and legacy antivirals, with effect-size estimates reflecting a balance between signal strength and accumulated clinical experience rather than simple report volume (Table 2).

To evaluate potential bias introduced by reducing multi-drug regimens to a single primary active drug, we performed a sensitivity analysis attributing phenotypes to all suspect drugs rather than a single primary active drug. Top-10 drug enrichment rankings were largely preserved across phenotypes, with 6–8 shared drugs per phenotype and Jaccard overlap ranging from 0.50 to 0.67 (Supplementary Table S6), indicating that primary drug attribution did not materially alter dominant drug–phenotype associations.

### 2.5. Polypharmacy and Network Centrality Are Strongly Associated with Serious Outcomes

**Figure 4:**
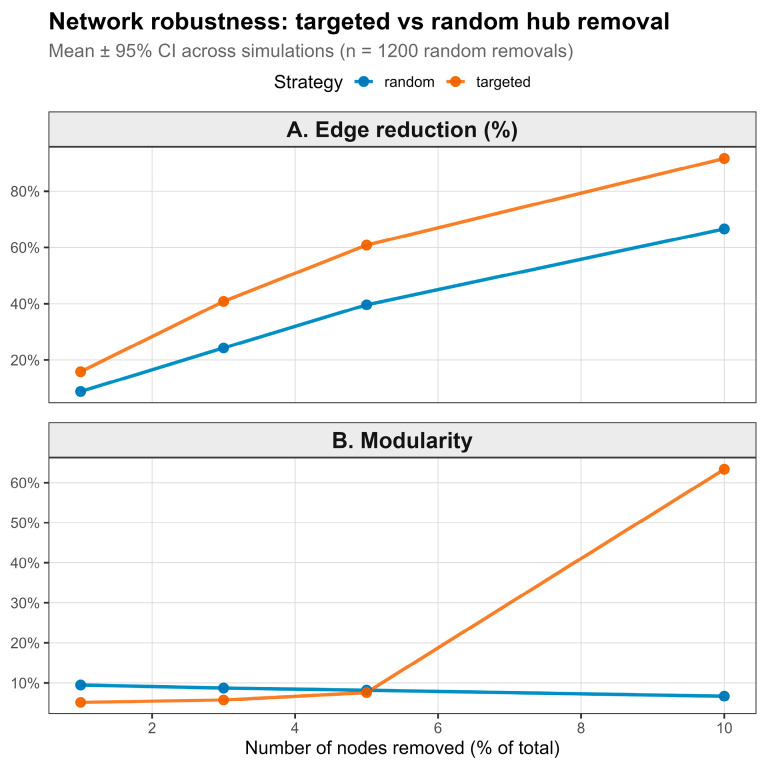
Network robustness under targeted versus random hub removal. (**A**) Percentage of edges removed as a function of the proportion of nodes eliminated. (**B**) Change in network modularity after node removal. Points and lines represent the mean across 1200 simulation replicates; error bars indicate 95% confidence intervals. Targeted removal corresponds to sequential deletion of highest-degree hubs, whereas random removal deletes nodes uniformly at random.

All regression models evaluating seriousness were performed at the ICSR (one-row-per-case) level to avoid pseudo-replication. Polypharmacy burden was associated with seriousness in ICSR-level models. Using total co-reported active burden as a single non-collinear predictor, each additional co-reported active drug increased the odds of a serious outcome by ~5% (OR 1.05, 95% CI 1.04–1.05, *p* = 4.11 × 10^−63^). Network robustness analyses supported a concentrated hub structure. Targeted removal of highly connected drugs produced substantially larger reductions in network connectivity and marked increases in modularity than random removal at every tested removal depth (Figure 4). With the removal of 10 nodes, targeted deletion increased modularity to more than 60% and eliminated nearly 80% of edges, whereas random deletion produced minimal modularity change and a more modest edge loss.

### 2.6. Integrated Mechanistic–System-Level Embedding Reveals Phenotype Associated Liability Structure

**Figure 5:**
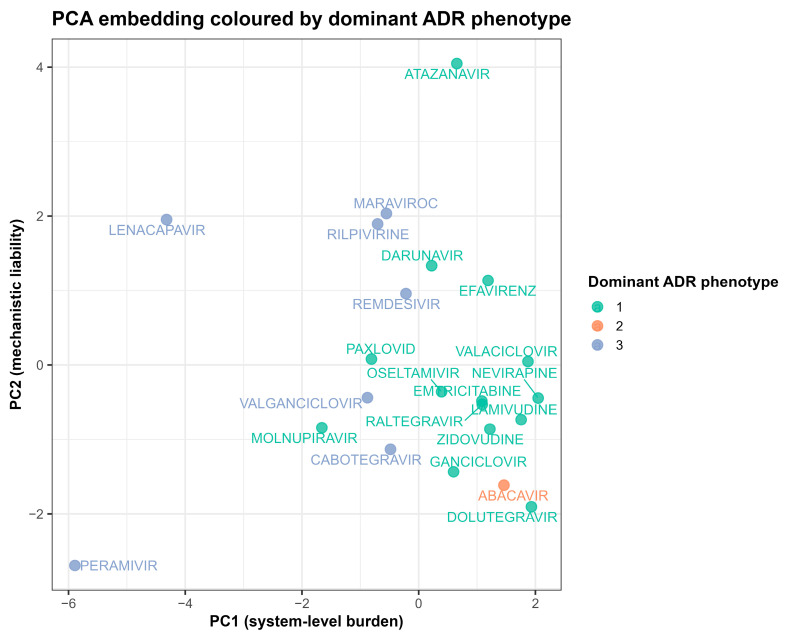
PCA embedding coloured by dominant ADR phenotype. Two-dimensional PCA projection of antivirals based on standardised ADMET, polypharmacy, and network metrics. Colours indicate the dominant ADR phenotype for each drug. Variable contribution and representation diagnostics are provided in Supplementary Figures S1 and S2; a complementary UMAP embedding is shown in Supplementary Figure S3.

Principal component analysis (PCA) of integrated ADMET, polypharmacy, and network features explained 67.3% of the variance in the first two components (PC1: 41.2%; PC2: 26.1%). Interpretation of variable loadings indicated that PC1 predominantly reflected system-level burden, driven by network connectivity and reporting breadth, whereas PC2 reflected intrinsic mechanistic liability, driven by predicted hepatic clearance, composite ADMET risk, and transporter/channel inhibition metrics (Figure 5; Supplementary Figures S1 and S2).

**Figure 6:**
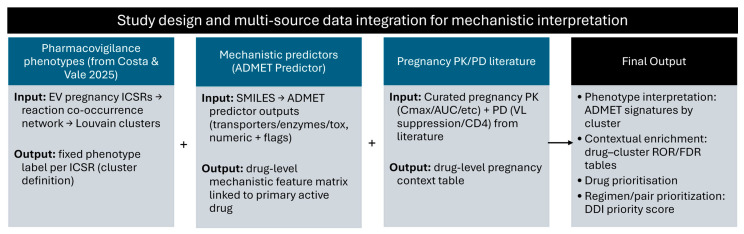
Study design and extended analytical workflow [21].

When coloured by dominant ADR phenotype, drugs associated with Cluster 3 showed preferential separation along the PC2 axis (Figure 6), consistent with an enriched transporter-related liability profile. Cluster-level ADMET signatures (Figure 2) demonstrated that Cluster 3 exhibited elevated predicted transporter inhibition signals, including the highest BSEP inhibition z-score among phenotypes (z = +1.16), alongside increased P-glycoprotein inhibition (z = +0.97) and moderately elevated composite ADMET and toxicity risk scores (z ≈ +0.73). These deviations occurred within a broader multidimensional transporter-liability context rather than reflecting a single dominant predictor. Variable contribution diagnostics are provided in the Supplementary Materials; a complementary UMAP embedding is shown in Supplementary Figure S3.

To assess clinical concordance, hepatobiliary-related MedDRA preferred terms (including cholestasis, bilirubin increase, and liver enzyme abnormalities) were evaluated across phenotypes. Cluster 3 was not characterised by over-representation of hepatobiliary adverse reactions (OR 0.48, 95% CI 0.38–0.60), indicating that elevated predicted BSEP inhibition reflects mechanistic vulnerability signals rather than a clinically defined hepatobiliary phenotype. Thus, the embedding supports a multidimensional transporter-related liability axis underlying the separation of Cluster 3, without implying direct organ-system specificity.

Leave-one-drug-out analyses demonstrated stable feature loadings for PC1 and PC2 (r > 0.90), indicating robustness of component interpretation. However, moderate sensitivity of the two-dimensional projection geometry was observed after removing influential drugs, consistent with the limited sample size (N = 25). Accordingly, embedding plots are interpreted as exploratory visualisations of multivariate similarity rather than as inferential cluster boundaries.

### 2.7. Composite Mechanistic Vulnerability Identifies High-Burden Drugs but No High-Priority Regimens

At the drug level, the mechanistic vulnerability index (MVI) demonstrated moderate concordance with empirical reporting burden and ADR phenotype breadth (Spearman ρ = 0.65) while remaining distinct from disproportionality estimates and simple reporting volume. Using the percentile-based MVI scale (Methods, Section 4.4.3), the highest-ranked drugs included dolutegravir (MVI_pct = 100.0), abacavir (95.8), and lamivudine (91.7) (Table 3).

The MVI integrates four dimensions: (i) phenotypic breadth, (ii) polypharmacy burden, (iii) network connectivity, and (iv) mechanistic predictors contributing to the Random Forest–derived high-burden probability. Mechanistic predictors include transporter inhibition signals (e.g., BSEP and P-gp), composite ADMET risk, and clearance-related features; however, the index does not assume one-to-one correspondence between individual ADMET predictors and specific ADR categories. Instead, it captures multidimensional system-level vulnerability reflecting interaction opportunity, regimen complexity, and intrinsic mechanistic liability.

Robustness diagnostics indicated that the ML component was not driven by any single drug. Leave-one-drug-out resampling yielded an AUC of 0.776, supporting the stability of discrimination across drugs. Variable importance rankings were moderately stable across resamples (coefficient of variation for top predictors 0.13–0.32). Removal of reporting volume and network centrality features (total_reports, mean_degree, mean_betweenness) did not alter model performance (AUC remained 0.583), indicating that the classifier was not driven by reporting intensity. Drug rankings were highly robust to alternative composite weight specifications (Spearman ρ = 0.997; Kendall τ = 0.969), supporting stability of the prioritisation framework. Variance inflation factors for RF probability, polypharmacy, and network features were all <2, indicating the absence of problematic multicollinearity (Supplementary Table S7). Robustness diagnostics for the ML component and composite ranking (N = 25 drugs), Supplementary Table S8.

Notably, several high-MVI drugs exhibited comparatively modest empirical ROR values, reinforcing that MVI captures system-level vulnerability (interaction opportunity and regimen complexity) beyond single drug–phenotype disproportionality. In particular, the consistent top ranking of dolutegravir reflects its role as a highly connected hub drug across pregnancy regimens and phenotypes, rather than implying maximal intrinsic toxicity. Conversely, drugs with lower MVI values generally showed reduced polypharmacy burden, fewer network connections, or more restricted phenotype involvement.

At the regimen level, application of the drug–drug interaction prioritisation score (DPS) did not identify any combinations meeting criteria for high or moderate priority under the pre-specified mechanistic thresholds; all evaluated regimens (*n* = 212) were classified as low priority (Table 4). This pattern suggests that, despite identifiable drug-level vulnerability signatures, commonly reported pregnancy antiviral regimens did not exhibit convergent multi-mechanism risk sufficient to warrant escalation under conservative prioritisation rules.

### 2.8. Exposure–Liability Simulations Under Pregnancy PK Bounds Support Select Mechanistic Interaction Hypotheses

Range-based simulations integrating literature PK bounds with ADMET potency parameters indicated that most drugs had a low probability of exceeding unity for BSEP-related exposure ratios under pregnancy conditions (P(ratio > 1) ≈ 0 for Cavg24/BSEP IC50 across evaluated drugs). In contrast, valaciclovir showed a substantial probability of exceeding unity for Cmax/BSEP IC50 (P(ratio > 1) = 0.688), consistent with higher peak exposure relative to the predicted BSEP inhibition threshold. For CYP-mediated ratios, efavirenz exhibited high exceedance probabilities for CYP1A2 and CYP2D6 (e.g., Cavg24/CYP1A2 Ki P(ratio > 1) = 1.000), supporting the mechanistic plausibility of interaction liability in pregnancy exposure ranges. Full ratio summaries are provided in Supplementary Table S9.

These exposure–liability simulations do not represent drug–drug interaction predictions or mechanistic PBPK modelling. Rather, they serve as plausibility checks to contextualise whether pregnancy-associated exposure shifts fall within ranges that could theoretically influence mechanistic vulnerability signals.

## 3. Discussion

In this study, we extended a previously validated pharmacovigilance network to mechanistically and pharmacometrically interpret pregnancy-related antiviral safety signals. By treating harmonised ADR clusters as stable phenotypic units and integrating ADMET-derived liabilities, polypharmacy, and network context, and pregnancy-specific PK/PD annotations, we evaluated whether pregnancy ADR signals can be mechanistically contextualised beyond disproportionality alone.

Harmonised ADR phenotypes exhibited marked heterogeneity in seriousness, polypharmacy burden, and mechanistic signatures, supporting the biological plausibility of phenotype-level differentiation. The high proportion of serious reports largely reflects regulatory classification rules in pregnancy pharmacovigilance, where congenital anomalies and Important Medical Events are inherently coded as serious in the underlying EudraVigilance variable, rather than de novo reclassification in this analysis. Contextual disproportionality analyses revealed cluster-specific enrichment patterns, with one phenotype (Cluster 3) showing a diverse and mechanistically coherent enrichment profile.

System-level features, particularly polypharmacy burden and network centrality, were strongly associated with serious outcomes. Fourth, unsupervised embedding separated drugs along axes reflecting system-level burden and intrinsic mechanistic liability, independently of outcome information. Finally, exposure–liability simulations incorporating pregnancy PK bounds supported select mechanistic interaction hypotheses without indicating widespread threshold exceedance under pregnancy exposure ranges.

Collectively, these findings suggest that pregnancy pharmacovigilance signals reflect an interplay between system-level complexity and drug-specific mechanistic liabilities, rather than isolated exposure effects.

### 3.1. Interpretation of Phenotype-Level Heterogeneity

The observation that harmonised ADR phenotypes differ substantially in the proportions of serious cases and ADMET signatures reinforces the premise that pregnancy-related adverse reporting can be structured rather than homogeneous. Notably, Cluster 1 combined high reporting volume with high seriousness, consistent with its broad representation of common antiretroviral regimens. In contrast, Cluster 3 exhibited lower overall seriousness but a distinct mechanistic profile and concentrated enrichment for specific antivirals. These findings extend prior work by demonstrating that ADR clusters derived from reaction co-occurrence are not merely statistical artefacts but map onto differentiable mechanistic contexts. The persistence of phenotype-specific ADMET patterns after harmonisation argues against residual confounding by report volume alone and supports the use of stable phenotypic units for downstream mechanistic interpretation.

### 3.2. Drug–Phenotype Enrichment as Contextual, Not Causal, Signals

In the present framework, Concordance was assessed qualitatively by examining whether drugs with strong disproportionality enrichment also displayed elevated mechanistic vulnerability scores or centrality within the co-medication network. Comparison of drug–phenotype enrichment with mechanistic and network-derived features revealed both concordant and discordant patterns. In some cases, drugs exhibiting phenotype enrichment also displayed elevated transporter or metabolic liability scores and high co-medication network connectivity, supporting a biologically plausible link between mechanistic properties, regimen complexity, and the observed reporting patterns. In other cases, enrichment signals occurred without corresponding mechanistic liability or exposure-related predictors, suggesting that contextual factors such as indication-specific use, novelty of reporting, or regimen composition may contribute to the observed signal. Drug–phenotype enrichment analyses identified a limited set of antivirals with strong cluster-specific associations, particularly within Cluster 3. These enrichments were interpreted in the context rather than as de novo signal discovery. According to the literature, integrase inhibitors and selected protease inhibitors have previously been the subject of pregnancy safety debate, lending external plausibility to the observed patterns [22,23]. At the same time, enrichment did not uniformly align with exposure magnitude or pregnancy-related dose adjustments. This decoupling underscores the limitations of disproportionality metrics when used in isolation and highlights the need for mechanistic overlays to interpret why certain drugs cluster within specific phenotypic profiles [24].

Within Cluster 3, the strongest enrichments for lenacapavir, maraviroc, rilpivirine, cabotegravir, and remdesivir likely reflect a mixture of indication-specific contexts (including newer agents and COVID-era antiviral use), reporting novelty, and regimen-level complexity rather than isolated drug toxicity. Importantly, the enrichment profile spans both emerging and legacy antivirals, supporting the interpretation that Cluster 3 captures a broader mechanistic phenotype in which transporter/metabolic liabilities interact with system-level exposure propagation through polypharmacy and network connectivity.

Taken together, these observations illustrate how mechanistic and network overlays can contextualise disproportionality signals, helping distinguish signals driven primarily by biological liability from those shaped by reporting dynamics or regimen structure. In this way, disproportionality metrics function not as standalone indicators of risk but as entry points for mechanistic interpretation within an integrated pharmacovigilance framework.

### 3.3. Polypharmacy and Network Effects as Central Drivers of Serious Outcomes

One of the most consistent findings across analyses was the strong association between polypharmacy burden and serious outcomes. In the case-level logistic regression model, each additional co-reported active drug increased the odds of seriousness by approximately 3% (OR 1.03, 95% CI 1.02–1.05). Although the effect size per drug is modest, the cumulative impact becomes meaningful in complex regimens. For example, an increase of five additional co-administered active drugs corresponds to an approximately 16% increase in the odds of a serious report (1.03^5^ ≈ 1.16). Meanwhile, a difference of ten drugs corresponds to an approximately 34% increase (1.03^10^ ≈ 1.34). Given that the median number of active drugs per report was 11 (IQR 6–27), this effect reflects the high regimen complexity commonly observed in pregnancy-related pharmacovigilance reports.

Network analyses further demonstrated that a small subset of highly connected drugs disproportionately contributed to network robustness, consistent with hub-driven exposure propagation. These hub drugs frequently appeared across multiple regimen combinations, increasing the opportunity for interaction propagation and shared phenotype reporting patterns. These results align with broader pharmacovigilance literature indicating that regimen complexity and interaction density are major contributors to adverse outcomes, particularly in vulnerable populations such as pregnant individuals [25,26,27]. Importantly, concomitant drugs were not treated as causal agents but as contextual amplifiers of system-level risk, consistent with pharmacovigilance conventions.

### 3.4. Mechanistic–System-Level Embedding Reveals Structured Liability Space

The unsupervised embedding analyses provide an outcome-independent view of how antivirals occupy a shared mechanistic–system-level space integrating ADMET, polypharmacy, and network features. The first two principal components explained 67.3% of total variance, indicating that a substantial proportion of inter-drug heterogeneity can be summarised along two orthogonal axes.

PC1 predominantly captured system-level burden, with high loadings for network connectivity and breadth of reporting. Drugs positioned toward the positive PC1 axis, therefore, reflect agents embedded within dense prescribing and interaction networks, often characterised by higher reporting volume and polypharmacy exposure. In contrast, PC2 reflected intrinsic mechanistic liability, driven by predicted hepatic clearance, composite ADMET risk, and transporter/channel inhibition metrics.

When coloured by the dominant ADR phenotype, drugs associated with Cluster 3 separated preferentially along PC2 rather than PC1. This pattern indicates that their grouping is not primarily explained by reporting intensity or network hubness, but instead by shared mechanistic properties. Cluster-level ADMET signatures demonstrated elevated predicted transporter inhibition signals, including the highest BSEP inhibition z-score among phenotypes (z = +1.16), increased P-glycoprotein inhibition (z = +0.97), and moderately elevated composite ADMET and toxicity risk scores (z ≈ +0.73). Importantly, these deviations occurred within a multidimensional transporter-related context rather than being attributable to a single dominant predictor.

Clinical concordance analyses further refined this interpretation. Despite elevated predicted BSEP inhibition, Cluster 3 was not characterised by over-representation of hepatobiliary adverse reactions (OR 0.48, 95% CI 0.38–0.60). This dissociation suggests that the embedding captures mechanistic vulnerability signals rather than a clinically manifest hepatobiliary phenotype, reinforcing the distinction between intrinsic liability architecture and observed organ-specific outcomes.

The concordance between PCA and the complementary Uniform Manifold Approximation and Projection (UMAP) supports the existence of a reproducible, structured liability space across both linear and nonlinear dimensionality reduction approaches. Leave-one-drug-out analyses demonstrated stable component loadings (r > 0.90), supporting robustness of axis interpretation. However, the moderate sensitivity of the two-dimensional projection geometry to the removal of influential drugs underscores the exploratory nature of the embedding, particularly given the limited sample size (N = 25). Accordingly, these visualisations are interpreted as representations of multivariate similarity rather than inferential cluster boundaries.

### 3.5. Exposure–Liability Ratios Under Pregnancy PK Bounds

Range-based exposure–liability simulations integrating pregnancy PK bounds with ADMET-predicted potency thresholds provided a quantitative check on biological plausibility. Across most evaluated drugs, BSEP-related ratios remained well below unity, even under conservative assumptions, arguing against widespread cholestatic liability driven by pregnancy exposure alone. However, select exceptions, valaciclovir for peak BSEP-related ratios and efavirenz for CYP-mediated ratios, provide concrete examples of how pregnancy exposure bounds can cross mechanistic potency thresholds in silico, motivating targeted follow-up in settings with hepatic vulnerability or high interaction burden.

These simulations did not indicate a class-wide amplification of risk. Instead, they differentiated a limited subset of agents with plausible interaction-mediated vulnerability from a background of mechanistically buffered profiles. While not implying clinical toxicity, the results support the plausibility of such vulnerabilities in specific mechanistic contexts. The simulations were designed for contextual validation, consistent with IVIVE principles, rather than deterministic risk prediction or formal PBPK analysis [28]. Instead, they functioned as plausibility checks to assess whether reported pregnancy-related exposure changes fall within ranges capable of influencing mechanistic vulnerability signals. Because gestational timing data were inconsistently reported in EudraVigilance ICSRs, trimester-stratified analyses were not performed. Literature-derived PK values (including trimester and postpartum data where available) were incorporated only for contextual benchmarking, and the resulting exposure–liability ratios should not be interpreted as trimester-resolved interaction estimates.

### 3.6. Implications for Pregnancy Pharmacovigilance and Mechanistic Prioritisation

No regimens exceeded the predefined high-priority DPS threshold. This likely reflects structured, guideline-concordant prescribing during pregnancy rather than an absence of mechanistic interaction potential. The DPS framework was designed for relative prioritisation and has not been externally calibrated. Consequently, classifications should be treated as hypothesis-generating rather than definitive DDI risk categories. The narrow dispersion of DPS values, without a bimodal pattern, further suggests the absence of a clearly segregated high-signal cluster.

The modest discrimination in the held-out data (AUC ≈ 0.58) and the limited number of drugs (N = 25) reinforce the view that the MVI is best understood as a prioritisation heuristic integrating multi-domain signals, rather than as a standalone predictive model. In this context, integrating pharmacovigilance data with mechanistic and pharmacometric annotations can move pregnancy safety assessment beyond binary signal detection toward structured, hypothesis-driven prioritisation. The absence of high-priority regimens in the DDI prioritisation underscores that pharmacological complexity and system-level vulnerability are not synonymous with prohibitive clinical risk, but instead require nuanced interpretation.

The percentile-based MVI should be interpreted as a relative measure of system-level vulnerability, capturing a drug’s tendency to appear across multiple ADR phenotypes under conditions of high polypharmacy burden and network connectivity, rather than as a surrogate for intrinsic teratogenicity or organ-specific toxicity. For example, dolutegravir’s consistent top ranking reflects its central role as a highly connected hub across pregnancy regimens and phenotypes, rather than implying exceptional intrinsic mechanistic hazard. Conversely, the comparatively modest empirical reporting odds ratios observed for several high-MVI drugs highlight that vulnerability in this framework arises from interaction opportunity and regimen context rather than disproportionality alone.

At the regimen level, the absence of Tier 1 or Tier 2 classifications reflects the composite DPS framework and threshold calibration. It is not driven solely by the Random Forest high-burden classifier, which contributes as one weighted component within the MVI. The absence of high- or moderate-priority combinations under the DPS framework does not indicate an absence of mechanistic vulnerability, but rather suggests that, within current pregnancy dosing practices and clinical monitoring, combined mechanistic liabilities for commonly reported antiviral regimens often remain below conservative prioritisation thresholds. This distinction reinforces the value of separating drug-level vulnerability profiling from regimen-level escalation decisions.

Methodologically, this framework provides a scalable approach for hypothesis generation in special populations where prospective trials are limited. Translationally, it supports targeted mechanistic follow-up rather than broad exposure-based concerns for drugs occupying high-vulnerability regions of the mechanistic–system-level space. From a regulatory and drug-development perspective, such an approach may support earlier and more structured benefit–risk evaluation for emerging antivirals entering clinical use in pregnancy, before robust outcome data are available.

### 3.7. Limitations

Several limitations should be considered when interpreting these findings. First, all pharmacovigilance analyses were based on spontaneous reporting data from EudraVigilance, which are inherently subject to underreporting, reporting bias, and incomplete covariate annotation. In particular, important covariates, including gestational timing, dosing adherence, indication severity, comorbidities, and exposure duration, were frequently unavailable or inconsistently recorded. Consequently, observed reporting patterns reflect reported associations rather than incidence rates or risk.

Second, mechanistic annotations were derived from one in silico ADMET predictor and curated literature summaries rather than direct in vivo measurements and should therefore be interpreted as indicative rather than quantitative. ADMET predictions are structure-based, not physiology-adjusted. Therefore, no pregnancy-specific clinical data on renal function, plasma-binding proteins, or fraction unbound were collected for inclusion. Only pregnancy PK integration captured physiological exposure, trimester-specific Cmax/AUC from clinical studies. Therefore, ADMET flags (transporter/CYP presence/absence) and polypharmacy metrics represent aggregate cohort exposure across gestation and are insensitive to trimester-specific physiological transitions (CYP3A4 induction, polypharmacy escalation).

Third, pregnancy pharmacokinetic data were sparse and heterogeneous, available for only a limited subset of drugs, and thus constrained quantitative extrapolation. The exposure–liability simulations were therefore constructed using literature-reported minimum–maximum bounds and uniform sampling assumptions. These simulations did not incorporate covariate-driven variability, trimester-specific population distributions, or uncertainty propagation. Accordingly, they serve as qualitative contextualisation tools rather than predictive models of foetal or maternal exposure risk.

Fourth, regimen complexity was drug-centric, not patient-centric. System-level metrics captured polypharmacy burden (median 11 actives/ICSR) and co-medication network topology exclusively from co-reported active substances. Patient-level covariates: comorbidities, gestational age, renal/hepatic function, and laboratory values were unavailable in public EudraVigilance exports. Multi-drug regimens were reduced to single primary active drugs via class hierarchy (INSTI > PI > NNRTI > NRTI > RdRp), preserving interpretability but underrepresenting regimen-level pharmacodynamic synergy/antagonism from boosters/backbones. Co-medication networks reflect co-reporting patterns rather than confirmed pharmacokinetic interactions, temporal sequencing, or exposure overlap. However, polypharmacy alone strongly predicted seriousness (OR 1.05 per additional active, 95% CI 1.04–1.05). Patient-stratified pharmacometric analyses incorporating hepatic/renal vulnerability would refine signals of vulnerability that have already been detected despite these conservative design choices.

Fifth, multivariate embeddings and machine-learning components were developed using a relatively small number of drugs (N = 25). Although leave-one-drug-out diagnostics demonstrated stable component loadings and ranking robustness, the geometric configuration of low-dimensional projections showed moderate sensitivity to influential agents. The mechanistic vulnerability index and DDI prioritisation scores are therefore relative, internally coherent ranking tools and were not calibrated to absolute clinical risk thresholds or externally validated datasets.

Importantly, this study does not estimate causal effects, individual-level risk, or comparative safety. The framework operates at the drug level and integrates mechanistic and pharmacovigilance-derived features specific to pregnancy-related EudraVigilance reports. Findings should not be interpreted as dosing recommendations, contraindication determinations, or regulatory safety classifications.

Finally, independent external validation was beyond the scope of this analysis. Comparable datasets that simultaneously contain harmonised pregnancy pharmacovigilance reports, trimester-aware exposure data, and mechanistic annotations remain limited. Future validation strategies may include replication using pregnancy-specific FAERS subsets, comparison with prospective pregnancy exposure registries, cross-database evaluation of mechanistic vulnerability rankings, and integration with trimester-stratified PBPK simulations. Such efforts would enable assessment of generalisability across regulatory systems and facilitate calibration of composite scoring thresholds.

### 3.8. Future Directions: From Mechanistic Interpretation to Clinical Translation and Generalisable Frameworks

This work was designed as a hypothesis-generating bridge between pregnancy pharmacovigilance and mechanistic modelling. Rather than producing clinical directives, it establishes a structured pathway for translating pharmacovigilance-derived signals into testable mechanistic hypotheses and modelling scenarios. Four forward applications emerge: (i) mechanistic prioritisation variables; (ii) structured PopPK/PBPK scenario design; (iii) contextual clinical interpretation; and (iv) methodological generalisation.

#### 3.8.1. Mechanistic Context Variables to Support Treatment Evaluation During Pregnancy

The mechanistic vulnerability index (MVI) and drug prioritisation score (DPS) provide structured summaries of multidimensional liability integrating polypharmacy burden, network connectivity, and mechanistic features. These scores are not calibrated to absolute clinical risk and are not intended to define contraindications. Instead, they function as comparative descriptors of systemic exposure context and mechanistic density.

High-percentile MVI rankings (e.g., dolutegravir, abacavir, lamivudine) reflect drugs embedded in dense prescribing and ADR networks rather than intrinsic toxicity. Such profiles identify agents for which interaction opportunities, exposure variability, or regimen complexity may warrant mechanistic scrutiny in subsequent modelling analyses.

Conversely, drugs demonstrating phenotype-specific enrichment or elevated exposure–liability plausibility ratios (e.g., efavirenz for CYP-mediated liability; valaciclovir for peak exposure relative to transporter thresholds) illustrate how pharmacovigilance signals can highlight mechanistic axes deserving targeted quantitative exploration.

#### 3.8.2. Translation into PopPK and PBPK Scenario Design Under Pregnancy-Specific Clinical Conditions

A key implication of this framework is that pharmacovigilance-derived mechanistic patterns can inform the design of pharmacometric models. Rather than relying on unguided sensitivity testing, the signals identified in mechanistic and network analyses can be used to propose candidate covariates that affect pharmacokinetic processes, including clearance, distribution, and absorption.

For example, signals related to CYP-mediated metabolism may indicate the need to evaluate covariates that affect intrinsic clearance. In contrast, transporter-related liabilities may motivate exploration of covariates that influence hepatic, renal, or placental transport processes. Similarly, system-level indicators such as polypharmacy burden or network connectivity may reflect regimen complexity and interaction potential, suggesting scenarios in which interaction-driven exposure variability should be examined.

In this way, pharmacovigilance-derived associations can be translated into testable hypotheses for PopPK or PBPK modelling, guiding the evaluation of covariate effects on exposure metrics such as C_max, AUC, or trough concentrations. This approach helps transform pharmacometric modelling from a primarily descriptive exercise into a hypothesis-driven and potentially predictive framework, particularly valuable in pregnancy populations where prospective pharmacokinetic data remain limited.

#### 3.8.3. Interpretive Clinical Implications

Although exploratory and drug-level in scope, several interpretive insights emerge. First, mechanistic burden appears to be influenced not only by intrinsic drug properties but also by regimen-level embedding within co-medication networks. This supports a regimen-aware conceptualisation of antiviral therapy during pregnancy.

Second, exposure–liability plausibility ratios suggest that pregnancy-induced pharmacokinetic shifts may interact with specific mechanistic axes (e.g., CYP metabolism, transporter inhibition). While not predictive of individual outcomes, such patterns can inform prioritisation of drugs for prospective PK investigation or registry enrichment.

Finally, distinguishing reporting-volume artefacts from biologically coherent multivariate structures may assist signal triage within regulatory pharmacovigilance workflows, particularly when pregnancy-specific trial data are limited.

#### 3.8.4. Generalisation Beyond Pregnancy and Antivirals

The modular structure of this framework enables adaptation to other vulnerable populations and therapeutic classes by substituting population-specific PK models, mechanistic predictors, and reporting systems while retaining composite scoring logic.

Pregnancy PK inputs could be replaced by neonatal, paediatric, or geriatric models; antivirals by anticonvulsants, oncology agents, or cardiovascular drugs; and EudraVigilance by FAERS or VigiBase datasets. Recalibration of the machine-learning layer within each context would preserve interpretability while maintaining population specificity.

More broadly, this approach positions pharmacovigilance not as a terminal safety signal, but as an entry point into structured mechanistic prioritisation and targeted modelling in populations where prospective experimentation is constrained.

## 4. Materials and Methods

### 4.1. Study Design and Multi-Source Data Integration Framework

This study extended a previously published network- and cluster-based pharmacovigilance framework for pregnancy-related antiviral safety analysis in EudraVigilance [21]. That prior work established the procedures for data extraction, reaction parsing, construction of reaction co-occurrence networks, community detection, and robustness assessment. These procedures were not re-estimated.

The present analysis integrated three complementary data layers, as illustrated in Figure 6: (A) Pharmacovigilance phenotypes (ICSR-level cluster labels); (B) Drug-level mechanistic predictors (ADMET features derived from SMILES inputs); (C) Pregnancy-specific PK/PD context curated from literature. These layers were linked through a hierarchical data-merging framework to enable mechanistic interpretation and drug prioritisation, to handle expanded ICSR structure. EudraVigilance exports include reaction- and drug-level expansions, which can introduce non-independence if rows are treated as independent observations. Expanded rows were used solely for mechanistic integration and mapping. All inferential analyses were conducted on deduplicated one-row-per-ICSR datasets grouped by EU.Local.Number. As a robustness check, regression models were refitted using cluster-robust standard errors clustered by ICSR identifier, yielding consistent estimates. System-level regimen complexity derived exclusively from co-reported active substances: polypharmacy burden (total unique actives/ICSR), pharmacokinetic boosters (presence/count), and co-medication network metrics (degree, betweenness centrality from within-ICSR drug co-occurrence).

Moreover, to facilitate reproducibility, a structured computational workflow describing data preprocessing, feature engineering, model specifications, and robustness diagnostics is provided in Supplementary Methods.

#### 4.1.1. Pharmacovigilance Data

Individual Case Safety Reports (ICSRs) submitted between January 2015 and June 2025 were retrieved from the EMA EudraVigilance public portal and restricted to spontaneous reports. Suspect drugs (reporter-designated causal candidates) and concomitant drugs (possible co-administration drugs) captured per standard pharmacovigilance convention (GVP Module VI). Reaction terms included maternal ADRs, foetal/neonatal outcomes, and pregnancy complications reported with antiviral exposure. As EudraVigilance exports expand ICSRs across reactions and drugs, two linked representations were constructed: (1) Deduplicated case-level datasets, one row per unique EU.Local.Number (N = 1938 pregnancy-related ICSRs), representing the true case-level denominator; (2) Expanded ICSR records (N = 17,058) after regimen parsing and active-substance linkage. Comorbidities, patient history (gestational age and trimester), and clinical covariates were unavailable in the EudraVigilance public exports. Patient demographics (age group, sex) were recorded but were not included in the complexity analysis.

Analyses sensitive to denominator inflation (seriousness proportions, polypharmacy summaries, regression models) were conducted at the deduplicated ICSR level. Analyses requiring drug-level linkage (mechanistic annotation, enrichment, network aggregation, scoring) were performed using the expanded table. A mapping between analytical blocks and dataset objects is provided in Supplementary Table S1.

Polypharmacy burden quantified by parsing suspect and concomitant medication lists into unique active ingredients for each ICSR. For each report, the number of interacting actives, concomitant actives, and total active substances was computed. In parallel, a co-medication network was constructed based on within-ICSR drug co-occurrence. Nodes represented active substances and edges represented co-reporting within the same ICSR. Network centrality measures (degree and betweenness) were calculated and subsequently aggregated at the drug level to capture system-level connectivity and interaction topology. Primary active drug assigned via class hierarchy (INSTI > PI > NNRTI > NRTI > RdRp) for mechanistic annotation. Co-medication network constructed from within-ICSR drug co-occurrence (nodes = actives, edges = co-reporting).

Concomitant characterised regimen complexity and network context. They were not treated as primary mechanistic drivers in drug-level scoring or in disproportionality analyses, which remained anchored to suspect drug reporting in accordance with pharmacovigilance conventions. Coverage across pharmacovigilance, ADMET, network, and pregnancy PK/PD domains in the analysis-ready dataset is summarised in Table 1.

Seriousness was defined using the EudraVigilance regulatory seriousness flag, aligned with International Council for Harmonisation of Technical Requirements for Pharmaceuticals for Human Use (ICH) E2A/E2D and Good Pharmacovigilance Practice (GVP) Module VI criteria. A report was classified as serious if it resulted in death, was life-threatening, required or prolonged hospitalisation, resulted in persistent disability, caused a congenital anomaly, or constituted an Important Medical Event.

A logistic regression model was fitted at the ICSR level (one row per EU.Local.Number) with regulatory seriousness as the binary outcome and total polypharmacy burden (poly_actives_total) as the primary predictor.

Sensitivity analyses were performed, restricting to female, non-follow-up, non-literature reports to evaluate potential reporting-structure artefacts.

#### 4.1.2. Mechanistic Annotation (ADMET Layer)

SMILES strings for each primary active drug were processed using ADMET Predictor v11.0 (Simulations Plus, Inc., Lancaster, PA, USA) to generate structure-based predictions of absorption, distribution, metabolism, excretion, and toxicity-related features, including transporter interactions, enzyme inhibition liabilities, and risk-based flags (e.g., CYP HLM intrinsic clearance, hERG pIC50, LogBB, human fraction unbound (hmufup), and composite risk scores). These were intrinsic drug properties, independent of physiological covariates. ADMET Predictor outputs were used to generate a harmonised set of structure-derived mechanistic descriptors across all drugs. These predictors were incorporated as relative indicators of transporter and enzyme interaction liability to enable consistent multivariate integration. They were not intended to replace experimentally established pharmacokinetic interaction data.

Because mechanistic annotations are defined at the active-substance level, a hierarchical primary active-drug assignment rule was applied to multi-drug regimens to enable linkage between ICSR-level observations and drug-level mechanistic features. The rule followed a predefined class priority (Integrase strand transfer inhibitors [INSTIs], Protease inhibitors [PIs], Non-nucleoside reverse transcriptase inhibitors [NNRTIs], Nucleoside reverse transcriptase inhibitors [NRTIs], and RNA-dependent RNA polymerase inhibitors [RdRp]). This dimensionality-reduction step was applied solely for mechanistic scoring and does not imply causal attribution of phenotype to a single regimen component.

ADMET outputs were aggregated into a drug-level mechanistic feature matrix and linked to the expanded ICSR-level table via the primary active drug identifier. Variable definitions and model outputs are detailed in Supplementary Table S4.

#### 4.1.3. Pregnancy PK/pd Contextual Annotation

Pregnancy PK parameters were curated from published clinical studies and include period-specific values (e.g., second trimester, third trimester, and postpartum). When multiple pregnancy-stage observations were available for a given drug, each stage-specific exposure estimate (Cmax and/or AUC0–24) was retained as reported in the source study [29,30,31,32,33,34,35,36,37,38,39,40,41,42,43,44,45,46] (Supplementary Tables S11A and S11B). No pooling across trimesters was performed during data curation.

PK/PD data were not used as predictors in clustering, disproportionality analyses, machine-learning models, or prioritisation score construction. Instead, they were used to contextualise mechanistic vulnerability signals, assess biological plausibility of phenotype-level enrichment, and conduct exploratory exposure–liability plausibility checks. These exposure–liability ratios do not represent drug–drug interaction predictions or PBPK modelling; they serve as contextual plausibility assessments.

### 4.2. ADR Phenotype Harmonisation

ADR phenotypes corresponded to previously derived reaction co-occurrence network communities. Clusters were not re-estimated. Analyses were performed at two levels: (i) ICSR-level, to quantify phenotype frequency, seriousness, and polypharmacy/network context; and (ii) drug-level, to aggregate mechanistic predictors and derive prioritisation scores. To ensure analytical stability and adequate mechanistic coverage, clusters representing <2% of reports were collapsed into a single “Other” category. The harmonised mapping was applied consistently to both the deduplicated and expanded datasets, yielding five phenotypes (Clusters 1, 2, 3, 5, and Other) (Supplementary Table S1).

### 4.3. Sensitivity Analyses, Disproportionality, Exploratory Machine Learning, and Missing Data

Disproportionality analyses were used as contextual annotations to characterise drug–phenotype enrichment rather than as primary signal discovery tools. For each drug–phenotype pair, 2 × 2 contingency tables were constructed, and reporting odds ratios (RORs) with Fisher’s exact tests were computed as described previously [21]. Associations were considered enriched for contextual interpretation at FDR-adjusted *p* < 0.05 whit ROR > 1.

Exploratory Random Forest analyses were performed to estimate high-burden probabilities used in the drug-level mechanistic vulnerability index (Section 4.4.3). Given the limited number of drugs (N = 25), the Random Forest component was treated as a ranking-oriented, hypothesis-generating signal rather than a calibrated predictive model.

Model stability was assessed using a 70/30 hold-out split (AUC = 0.583), leave-one-drug-out (LODO) resampling (AUC = 0.776), and out-of-bag (OOB) error diagnostics (OOB error = 0.133). Because the high-burden class is sparse at the drug level, AUC-based resampling metrics were prioritised over overall misclassification error for interpretation.

Uncertainty in predicted probabilities was quantified via bootstrap resampling (B = 500), reporting 95% percentile intervals (median CI width 0.345; IQR 0.369; range 0.088–0.863.

Additional robustness safeguards included variable importance stability assessment, removal of reporting-volume and network centrality features, and evaluation of alternative composite weight specifications. To evaluate potential reporting-structure artefacts, we performed a sensitivity analysis restricting the dataset to female, non-follow-up (Parent.Child.Report = No), non-literature reports. This restriction retained 784 of 1938 ICSRs (40.5%) and yielded similar effect estimates.

Unsupervised dimensionality reduction (PCA/UMAP) was used to characterise mechanistic–system-level similarity independent of outcomes (Section 4.4.5).

No imputation was applied for mechanistic or PK/PD domain coverage analyses. To generate RF-predicted probabilities across drugs, missing predictor values were median-imputed within predictor columns, allowing scoring of drugs with partial mechanistic annotation.

All analyses were performed in R (version 4.2.2) on Windows 10 64-bit. Key packages included tidyverse (v2.0.0), igraph (v1.4.1), ranger (v0.15.1), pROC (v1.18.0), pheatmap (v1.0.12), FactoMineR (v2.7), and factoextra (v1.0.7). Generative artificial intelligence (GenAI) tools were used to assist with text drafting and editing, methodological clarification, and code development during data analysis. GenAI did not generate data, perform independent analyses, or make scientific decisions. All analyses, interpretations, and conclusions were conducted and validated by the authors.

### 4.4. Mechanistic Vulnerability Scoring, Exposure Thresholds, and DDI Prioritisation

To integrate pharmacovigilance burden, mechanistic liability, and system-level interaction context into a unified framework, complementary prioritisation outputs were constructed at the drug, regimen, and drug-pair levels. These outputs were designed as hypothesis-generating prioritisation tools to support mechanistic interpretation and identify antivirals and combinations warranting further investigation, rather than to estimate individual-level risk or clinical outcomes.

Mechanistic and pharmacometric data were available in varying degrees across domains. ADMET-based mechanistic annotations were available for 22 of 25 drugs in the analysis-ready dataset. Pregnancy PK metrics were retrieved for 15/25 drugs. PD endpoints (virologic suppression, CD4 recovery) were available for the six-drug subset with clinical PK data. PD integration is restricted to exposure-liability simulations. No PD data extrapolation across drugs. Analyses integrating each domain were restricted to drugs with available information; no cross-drug extrapolation was performed.

#### 4.4.1. Mechanistic Feature Standardisation

Continuous ADMET and PK parameters were standardised using z-scores. Binary indicators capturing transporter and enzyme liabilities (including P-gp, BSEP, and CYP inhibition flags) were encoded as categorical variables (presence/absence).

#### 4.4.2. Exposure–Liability Ratios

Because gestational timing was not available at the ICSR level, trimester-stratified pharmacovigilance analyses were not possible, and no trimester-specific physiology model (e.g., dynamic enzyme activity scaling) was implemented. Accordingly, exposure–liability ratios represent pregnancy-contextual plausibility checks based on literature-reported pregnancy-stage exposures, rather than trimester-resolved interaction predictions. These metrics were used for contextual validation and were not applied as deterministic decision rules or predictors in model construction.

For drugs with literature-reported pregnancy PK bounds (Cmax and/or AUC_0–24_) and ADMET Predictor–derived potency parameters (BSEP IC50 and/or CYP Ki; µM), a range-based Monte Carlo procedure was used to propagate uncertainty in pregnancy exposure within reported minimum–maximum bounds. PK metrics were converted from µg/mL to µM using molecular weight; AUC-based average concentration was computed as Cavg_24_ = (AUC_0–24/24_). For each drug and each available pregnancy-stage row, exposures were sampled uniformly within literature-reported minimum and maximum bounds. When bounds were unavailable, the reported central estimate was used. For each ratio (e.g., Cmax/IC50, Cavg24/Ki), the median, 95% interval, and exceedance probabilities P(ratio > 1) and P(ratio > 10) were computed. Full implementation details and results are provided in the Supplementary Material.

Ratios exceeding unity were interpreted as indicating the mechanistic plausibility of exposure approaching or exceeding the selected potency threshold, consistent with standard IVIVE margin concepts. These ratios were not treated as definitive DDI predictions and were not used as predictors in clustering, regression, Random Forest modelling, or DPS thresholding.

#### 4.4.3. Drug-Level Mechanistic Vulnerability Index (MVI)

An MVI was constructed as a relative prioritisation score to integrate pharmacovigilance burden, mechanistic liability, and system-level interaction context. MVI was designed for hypothesis generation and ranking, not estimation of absolute clinical risk.

First, an empirical burden–breadth proxy was defined for each drug as the product of reporting volume and phenotypic breadth:

(1) $${\text{adr\_composite}}_{d}={\text{total\_reports}}_{d}\times n_{clusters,d}.$$

A binary high-burden label was then assigned using the upper quartile of the empirical burden distribution:

(2) $${HighBurden}_{d}=\left\{\begin{array}{cc} 1, & \mathrm{if}\;{\text{adr\_composite}}_{d}\geq Q_{0.75},\\ 0, & \mathrm{otherwise}. \end{array}\right.$$

A Random Forest classifier was trained to estimate the probability of high empirical burden:

(3) $${\hat{P}}_{d}=Pr\left({HighBurden}_{d}=1|X_{d}\right),$$

where $X_{d}$ comprised drug-level aggregates of polypharmacy metrics, network centrality measures, ADR phenotype features, and ADMET-derived mechanistic predictors.

The composite raw vulnerability score was defined as a weighted combination of the predicted high-burden probability and standardised system-level components:

(4) $${\text{composite\_raw}}_{d}=0.60\;{\hat{P}}_{d}+0.15\;z({polypharmacy}_{d})+0.15\;z({network}_{d})+0.10\;z({\text{cluster\_severity}}_{d}),$$

where z(⋅) denotes z-score standardisation.

Because the objective was ranking rather than absolute calibration, the primary MVI reported in the manuscript was expressed on a 0–100 percentile scale to improve interpretability and reduce sensitivity to single extreme values:

(5) $${MVI}_{d}=100\times \text{percent\_rank}\;\left({\text{composite\_raw}}_{d}\right).$$

MVI outputs were evaluated for internal coherence with ADR phenotype seriousness, reporting breadth, and contextual disproportionality measures. Sensitivity checks using alternative monotone normalisations produced identical drug ordering, supporting the robustness of the prioritisation. To evaluate potential redundancy between the RF-derived probability and explicitly weighted system-level components, we computed pairwise correlations and variance inflation factors (VIF). Correlations were moderate (r = 0.65 between RF probability and polypharmacy; r = 0.41 with network burden), and VIF values were low (<1.6), indicating no problematic multicollinearity.

#### 4.4.4. Regimen-Level Drug–Drug Interaction Prioritisation Score (DPS)

A regimen-level DPS was constructed to summarize combinations based on mechanistic overlap, network amplification, and safety vulnerability. The DPS integrated: (i) mechanistic overlap (MOS), (ii) network amplification (NAS), and (iii) safety vulnerability (SVS). DPS outputs were stratified into prioritisation tiers (high, moderate, low) using percentile-based thresholds. Because the high-burden label was defined as the upper quartile of the composite score, resulting in approximately 75:25 class imbalance (N = 25 drugs), minority-class performance metrics were evaluated under leave-one-drug-out resampling. Using a 0.5 threshold, precision, recall, and F1-score for the high-burden class were 0.25, 0.20, and 0.22, respectively.

#### 4.4.5. Unsupervised Mechanistic Embedding

To explore structured mechanistic and system-level groupings independently of outcomes, PCA and UMAP were applied to z-standardised drug-level features capturing polypharmacy burden, network topology (mean degree, mean betweenness), reporting breadth (total reports, number of clusters), and mechanistic liability (ADMET_Risk_mean, BSEP_IC50_mean, hERG_pIC50_mean, CYP_HLM_CLint_mean). No outcome, seriousness, disproportionality, or prioritisation variables were included. PCA was conducted exclusively on continuous ADMET predictors after z-score normalisation. Binary flag variables were not included in PCA and were displayed only for descriptive comparison in the heatmap visualisation. UMAP was used as a complementary nonlinear visualisation.

## 5. Conclusions

In summary, pregnancy-related antiviral safety signals in spontaneous reporting data organise into reproducible ADR phenotypes shaped by the interplay of intrinsic mechanistic liability (ADMET/PK-informed features) and system-level regimen complexity (polypharmacy and network context). By integrating pharmacovigilance networks with in silico ADMET liabilities and curated pregnancy PK annotations, we provide an interpretable, hypothesis-generating framework to contextualise residual signals without conflating disproportionality with causality. This phenotypic–mechanistic mapping enables pragmatic prioritisation for follow-up, highlighting drugs and scenarios where targeted pharmacometrics modelling, interaction-aware monitoring, or prospective pregnancy studies may be most informative, thereby advancing more mechanistically grounded pharmacovigilance in populations historically underrepresented in clinical trials.

## Figures and Tables

**Table 1:** The study dataset used feature coverage for mechanistic integration.

Domain	Metric	Value
Case-level data	Unique pregnancy-related ICSRs	1938
	Serious ADR proportion (case-level)	0.92
Reaction-level representation	Expanded ICSR records (drug–ADR pairs; same 1938 cases structurally expanded)	17,058
Drug-level coverage	Unique drugs included in drug-level analyses	25
ADR phenotypes	Harmonised phenotype clusters	5
	Proportion assigned to “Other” cluster	0.02
Polypharmacy	Median polypharmacy burden (IQR)	11 (6–27)
	Proportion of ICSRs with ≥5 concomitant actives	0.20
Network topology	Median network degree (IQR)	43.8 (40.6–52.4)
	Median network betweenness (IQR)	0.25 (0.22–0.30)
ADMET coverage	Drugs with ADMET_Risk available	22/25
	Drugs with BSEP IC50 available	22/25
	Drugs with hERG pIC50 available	22/25
	Drugs with CYP HLM intrinsic clearance available	22/25
PK/PD integration	Drugs linked to pregnancy PK parameters	15/25
	Drugs with viral load suppression endpoints	6/25

**Table 2:** Top 10 drugs enriched in ADR phenotype Cluster 3 (ROR analysis).

Drug	Cluster 3 ROR	FDR q-Value	Total Reports (Drug)
Lenacapavir	14.20	1.2 × 10^−4^	10
Maraviroc	4.70	2.0 × 10^−7^	51
Rilpivirine	4.00	1.5 × 10^−12^	119
Cabotegravir	3.81	1.7 × 10^−8^	82
Remdesivir	3.23	1.3 × 10^−11^	159
Valganciclovir	2.68	1.5 × 10^−3^	54
Oseltamivir	2.37	9.8 × 10^−10^	259
Atazanavir	2.12	3.1 × 10^−20^	812
Efavirenz	1.71	1.6 × 10^−14^	1200
Darunavir	1.54	3.3 × 10^−8^	1009

**Table 3:** Top-ranked drugs by mechanistic vulnerability index. Values shown are percentile-based MVI (0–100) derived from the composite vulnerability score (Methods, Section 4.4.3). Empirical ROR is provided for contextual comparison and corresponds to the drug’s dominant ADR phenotype (mode of harmonised phenotype assignments).

Drug	MVI (0–100)	RF P (High Burden)	Serious ADR Proportion	Total Reports	ADR Phenotypes (n)	Mean Network Degree	Mean Polypharmacy	Dominant Phenotype	Empirical ROR *
Dolutegravir	100.0	0.778	0.91	1761	5	57.83	31.09	1	0.08
Abacavir	95.8	0.366	0.96	2334	5	52.36	39.72	2	0.11
Lamivudine	91.7	0.756	0.93	1809	5	54.53	18.47	1	0.08
Ganciclovir	87.5	0.384	0.92	132	5	55.43	14.88	1	0.01
Zanamivir	83.3	0.082	1.00	7	2	91.57	5.43	1	<0.001
Emtricitabine	79.2	0.386	0.88	536	5	49.21	14.67	1	0.02
Zidovudine	75.0	0.264	0.93	1408	5	48.74	14.60	1	0.06
Nevirapine	70.8	0.200	0.97	1151	5	56.96	11.32	1	0.05
Valaciclovir	66.7	0.096	0.88	242	5	66.26	6.83	1	0.01
Raltegravir	62.5	0.166	0.93	1500	5	43.07	14.05	1	0.07

* Empirical ROR corresponds to the drug’s dominant phenotype.

**Table 4:** Distribution of drug–drug interaction prioritisation tiers.

Priority Tier	Number of Regimens	Proportion (%)
Tier 1—High	0	0
Tier 2—Moderate	0	0
Tier 3—Low	212	100

## Data Availability

The original contributions presented in this study are included in the article/Supplementary Materials. Further inquiries can be directed to the corresponding author.

## Supplementary Materials

The following supporting information can be downloaded at: https://www.mdpi.com/article/10.3390/ph19030450/s1, Table S1. Unit of analysis, dataset, and denominators used across analytical blocks; Table S2. Mapping of original ADR clusters to harmonised phenotypes; Table S3. Serious outcome proportions by harmonised ADR phenotype; Table S4. Between-phenotype differences in ADMET predictors and liability flags; Table S5. Summary of significant drug–phenotype enrichment by harmonised phenotype; Table S6. Robustness of drug–phenotype enrichment to attribution strategy; Table S7. Assessment of redundancy among MVI components; Table S8. Robustness diagnostics for the ML component and composite ranking; Table S9. Range-based simulation of pregnancy exposure–liability ratios; Table S10. Definitions of engineered variables, PCA inputs, and ADMET predictors; Table S11A. Pregnancy PK/PD data sources; Table S11B. Pregnancy PK/PD variable definitions; Figure S1. PCA variable contributions to PC1 and PC2; Figure S2. PCA cos^2^ (quality of representation); Figure S3. UMAP embedding (exploratory) [29,30,31,32,33,34,35,36,37,38,39,40,41,42,43,44,45,46].

## Abbreviations

The following abbreviations are used in this manuscript:

ADR	Adverse Drug Reaction
ADMET	Absorption, Distribution, Metabolism, Excretion, and Toxicity
PK/PD	Pharmacokinetics/Pharmacodynamics
ICSR	Individual Case Safety Report
EMA	European Medicines Agency
ROR	Reporting Odds Ratio
FDR	False Discovery Rate
OR	Odds Ratio
CI	Confidence Interval
PCA	Principal Component Analysis
UMAP	Uniform Manifold Approximation and Projection
MVI	Mechanistic Vulnerability Index
DDI	Drug–Drug Interaction
DPS	Drug–Drug Interaction Prioritisation Score
BSEP	Bile Salt Export Pump
CYP	Cytochrome P450
hERG	Human Ether-à-go-go–Related Gene (potassium channel)
IC_50_	Half-maximal Inhibitory Concentration
K_i_	Inhibition Constant
AUC	Area Under the Concentration–Time Curve
Cmax	Maximum Plasma Concentration
Cavg(24)	Average Plasma Concentration over 24 Hours
IVIVE	In Vitro to In Vivo Extrapolation
INSTI	Integrase Strand Transfer Inhibitor
PI	Protease Inhibitor
NNRTI	Non-Nucleoside Reverse Transcriptase Inhibitor
NRTI	Nucleoside Reverse Transcriptase Inhibitor
RdRp	RNA-Dependent RNA Polymerase
PK-Sim	Physiologically Based Pharmacokinetic Simulation Platform
PBPK	Physiologically Based Pharmacokinetic
PopPK	Population Pharmacokinetic
FAERS	FDA Adverse Event Reporting System
EMR	Electronic Medical Record
AI	Artificial Intelligence
ML	Machine Learning
