# Supplementary material for: From Pharmacovigilance Signals to Mechanistic Phenotypes: Integrating ADMET, PK/PD, and Network Context to Interpret Antiviral Safety in Pregnancy
Source: Pharmaceuticals (Basel). 2026 Mar 11;19(3):450. doi: 10.3390/ph19030450 (PMC13029279; doi:10.3390/ph19030450)
Supplement: Supplementary file 1 [file pharmaceuticals-19-00450-s001.zip › pharmaceuticals-4167839-supplementary.pdf]

Supplementary Materials This supplement provides extended documentation of data harmonisation, variable construction, statistical testing, and exploratory analyses supporting the mechanistic interpretation presented in the main manuscript. All mechanistic, network, and pharmacokinetic annotations are intended for hypothesis generation and prioritisation rather than causal inference or clinical risk estimation. References to previously published clustering methodology are provided where relevant.

Table S1. Unit of Analysis, Dataset and Denominators Used Across Analytical Blocks

Analytical Block	Unit of Analysis	Dataset Description	Denominator (N)
Core pregnancy case identification	ICSR	Deduplicated pregnancy-related EudraVigilance ICSRs (one row per EU.Local.Number)	1,938 unique ICSRs
Phenotype assignment (cluster mapping)	ICSR	Each ICSR assigned to a dominant ADR cluster (cluster2)	1,938 ICSRs
Expanded mechanistic integration table	ICSR-level record	Reaction-level expansion of the same 1,938 ICSRs after regimen parsing and primary active drug assignment	17,058 records
Phenotype frequency distribution (Figure 1)	Record mapped to phenotype	Distribution of phenotype labels across expanded records	17,058 records
Seriousness proportion (overall & by phenotype)	ICSR	Case-level seriousness (EudraVigilance seriousness flag)	1,938 ICSRs
Polypharmacy burden summaries	ICSR	Number of active substances per ICSR	1,938 ICSRs
Seriousness ~ Polypharmacy regression	ICSR	Logistic regression model predicting seriousness	1,938 ICSRs
Drug–phenotype enrichment (ROR/Fisher tests)	Drug–phenotype record	Drug–phenotype contingency counts	17,058 records
Drug-level ADMET summaries	Drug	Aggregated drug-level ADMET metrics	25 drugs (subset with available data)

Analytical Block	Unit of Analysis	Dataset Description	Denominator (N)
Phenotype-level ADMET profiles	Record mapped to phenotype	ADMET distributions per phenotype	e.g., 12,049 records in Cluster 1
Mechanistic Vulnerability Index (MVI)	Drug	Integrated score combining ADMET, PK/PD, enrichment, and network context	25 drugs

Original ADR clusters derived from reaction co-occurrence network analysis (Costa & Vale, 2025) were harmonised post hoc to ensure statistical stability and adequate coverage of mechanistic and pharmacokinetic annotations. Clusters representing fewer than 2% of pregnancy-related analysis records in the original clustering dataset were collapsed into a single "Other" category. No re-clustering or modification of the original community detection procedure was performed.

Supplementary Table S2. Mapping of original ADR clusters to harmonised phenotypes used for mechanistic analyses. Clusters with <2% of reports were collapsed due to low frequency. S1 counts come from the original clustering dataset (full pregnancy-expanded record set).

Original cluster	Number of reports (n)	Proportion of pregnancy reports (%)	Harmonised phenotype
1	14,295	58.58	Cluster 1
2	3,503	14.36	Cluster 2
3	4,723	19.35	Cluster 3
4	100	0.41	Other
5	1,370	5.61	Cluster 5
6	179	0.73	Other
7	189	0.77	Other
8	43	0.18	Other

Following harmonisation, five analytically stable ADR phenotypes were retained for downstream analyses: Clusters 1, 2, 3, 5, and a low-frequency "Other" category. The collapsed "Other" phenotype comprised 511 ICSRs (2.09% of pregnancy-related reports). Harmonisation was performed solely to stabilise phenotype denominators for mechanistic,

network, and pharmacokinetic integration and did not alter the original cluster definitions or reaction compositions reported previously.

Serious outcome proportions were calculated for each harmonised ADR phenotype using the binary seriousness indicator defined in the main Methods. Values are reported as the number of phenotype-associated ICSR-level records and the proportion classified as serious. This table supports the main-text seriousness comparisons across phenotypes.

Supplementary Table S3. Serious outcome proportions by harmonised ADR phenotype

Harmonised phenotype	Number of reports (n)	Serious outcome proportion
1	10,314	0.981
2	2,102	0.874
3	3,272	0.770
5	1,010	1.000
Other	360	0.636

Between-phenotype differences in ADMET predictors were evaluated across harmonised ADR phenotypes. Continuous ADMET variables were tested using non-parametric omnibus tests, and binary liability indicators were assessed using contingency-based tests. Reported p-values were adjusted for multiple testing using the Benjamini–Hochberg procedure. This table supports the statement that ADR phenotypes exhibit strong and systematic mechanistic heterogeneity.

Supplementary Table S4. Between-phenotype differences in ADMET predictors and liability flags

ADMET variable	Test statistic	df	p-value	FDR-adjusted p
ADMET_Risk	3626	4	$<1\times10^{-300}$	$<1\times10^{-300}$
BSEP_IC50	180	4	6.04×10^{-38}	9.06×10^{-38}
CYP_HLM_CLint	188	4	1.19×10^{-39}	1.92×10^{-39}
HEP_hCLint	1813	4	$<1\times10^{-300}$	$<1\times10^{-300}$
HEP_mCLint	489	4	1.96×10^{-104}	3.74×10^{-104}
HEP_rCLint	1814	4	$<1\times10^{-300}$	$<1\times10^{-300}$
LogBB	48.1	4	8.93×10^{-10}	8.93×10^{-10}
MUT_Risk	2344	4	$<1\times10^{-300}$	$<1\times10^{-300}$
MUTx_Risk	1787	4	$<1\times10^{-300}$	$<1\times10^{-300}$
S.MDCK	1178	4	9.70×10^{-254}	2.55×10^{-253}
…11 additional variables				

Supplementary Table S5. Summary of significant drug-phenotype entichment by harmonized phenotype. Phenotype 1 represented the dominant reporting phenotype and showed broad enrichment for multiple high-frequency antiretrovirals, consistent with its large report base. Phenotype 2 displayed strong but concentrated enrichment for abacavir, suggesting phenotype anchoring rather than diffuse risk. Phenotype 3 exhibited the most diverse enrichment profile, involving multiple antivirals with moderate-to-strong RORs, and was therefore prioritised for mechanistic interpretation in the main analysis. Phenotype 5 showed pronounced enrichment for dolutegravir and abacavir, consistent with regimen-specific ADR patterning. The "Other" phenotype aggregates several low-frequency clusters and should be interpreted cautiously due to heterogeneity and limited report counts.

**Phenotype (cluster2)**	**Total reports (n_cluster2)**	**No. enriched drugs (q < 0.05 & ROR > 1)**	**Top enriched drugs (by ROR)**	**Median ROR (enriched drugs)**
1	14,295	High (>15)	Nevirapine, Zidovudine, Raltegravir	~1.8–2.1
2	3,503	Moderate (~6–8)	Abacavir, Valganciclovir	~2.0–3.0
3	4,723	High (>10)	Lenacapavir, Maraviroc, Rilpivirine	~2.5–4.0
5	1,370	Low–moderate (~4–6)	Dolutegravir, Abacavir	~3.0–5.0
Other	511	Low (~3–4)	Tenofovir, Efavirenz	~2.0–3.0

Drug–phenotype enrichment analysis for all antiviral agents across harmonised ADR phenotypes. For each drug–phenotype pair, enrichment was assessed using contingency table–based disproportionality metrics. Reported statistics include raw counts, reporting odds ratios (ROR), proportional reporting ratios (PRR), Fisher's exact test p-values, and Benjamini–Hochberg–adjusted q-values. Results are provided as contextual annotations to support mechanistic interpretation rather than as primary signal detection.

Supplementary Table S6. Robustness of Drug–Phenotype Enrichment to Attribution Strategy. Enrichment rankings under primary attribution were compared with all-suspect attribution. Overlap indicates robustness of drug–phenotype associations to attribution strategy.

**Cluster**	**Shared Top-10 Drugs**	**Jaccard**
1	7	0.538
2	7	0.538
3	7	0.538

Cluster	Shared Top-10 Drugs	Jaccard
5	6	0.500
Other	8	0.667

Supplementary Figure S1 — PCA variable contributions to both PC1 and PC2

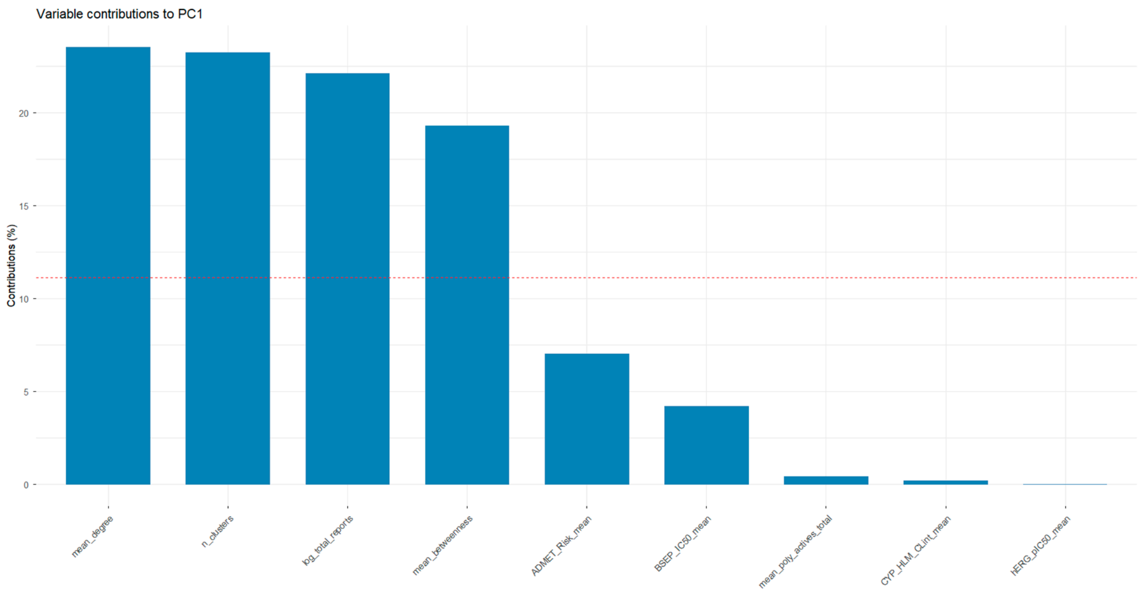

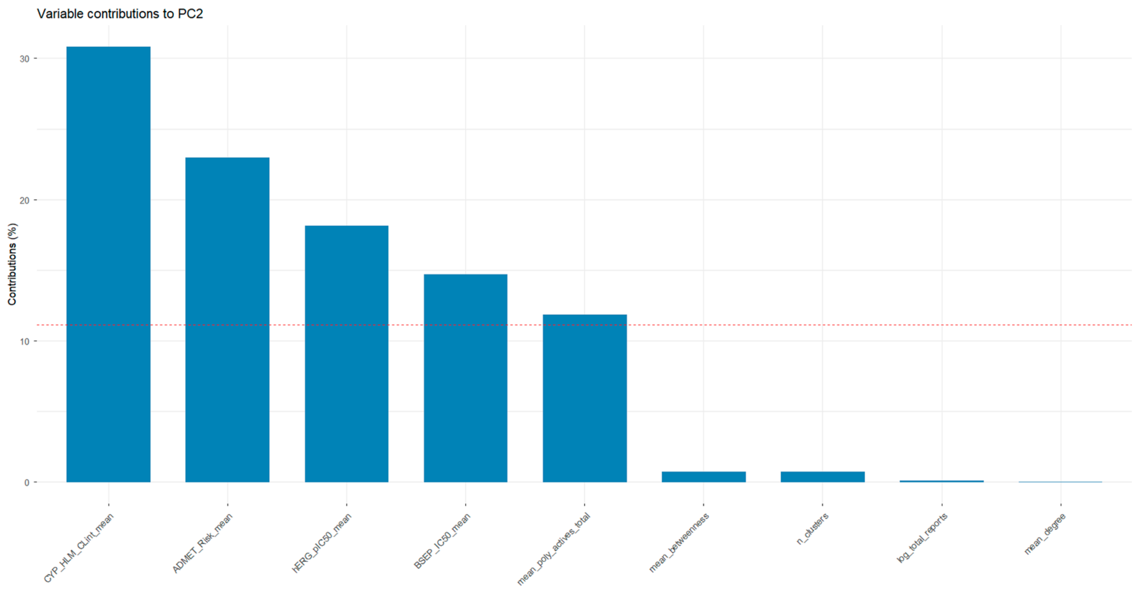

Supplementary Figure S2 — PCA $\cos^2$ (quality of representation)

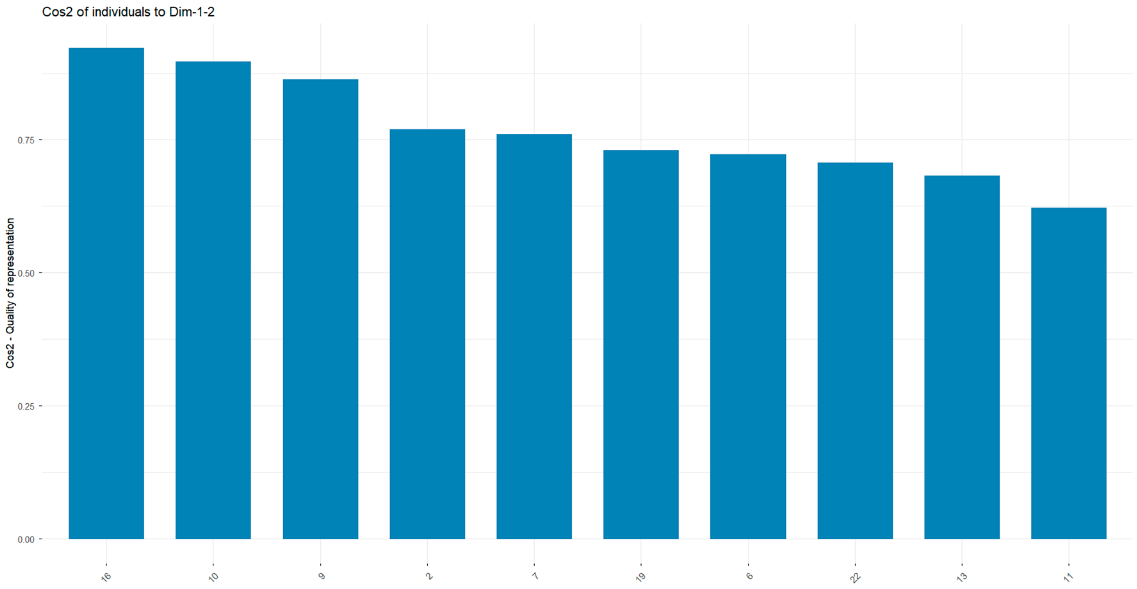

Supplementary Figure S3 — UMAP embedding (exploratory)

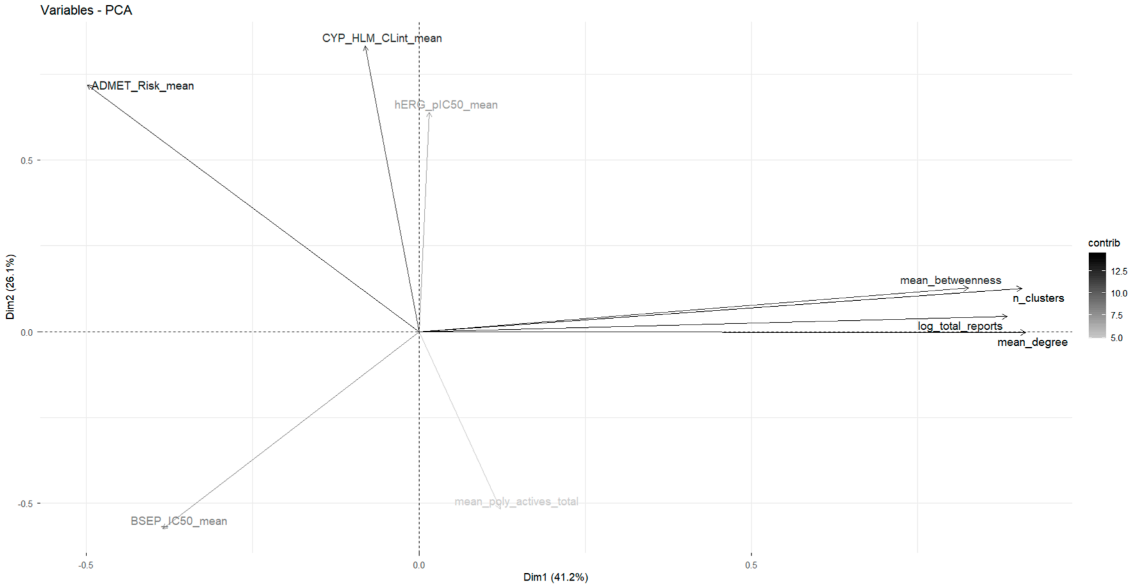

Supplementary Table S7. Assessment of redundancy among MVI components (N = 26 drugs) with correlation matrix

Component	RF_prob_highrisk	poly_z	network_z	ADMET_Risk_z
**RF_prob_highrisk**	1.000	0.652	0.411	-0.529
**poly_z**	0.652	1.000	0.051	-0.419
**network_z**	0.411	0.051	1.000	-0.423
**ADMET_Risk_z**	-0.529	-0.419	-0.423	1.000

Pearson correlation coefficients among RF-derived probability and explicitly weighted domain components. To further assess potential multicollinearity among MVI components, variance inflation factors (VIFs) were computed from a linear model with RF-derived high-risk probability as the outcome and poly_z, network_z, and ADMET_Risk_z as predictors. VIF values were 1.24 for poly_z, 1.25 for network_z, and 1.51 for ADMET_Risk_z. All values were well below the conventional threshold of 3, indicating absence of problematic multicollinearity and supporting the independent contribution of each domain in the composite index.

Supplementray Table S8. Robustness diagnostics for the ML component and composite ranking (N = 25 drugs).

The Random Forest component demonstrated stable discrimination under leave-one-drug-out resampling (AUC = 0.776). Removal of reporting volume and network centrality features did not alter model performance (AUC unchanged at 0.583), indicating independence from

reporting intensity. Variable importance rankings showed moderate stability across resamples (CV 0.13–0.32). Drug-level composite rankings were highly robust to alternative weight specifications (Spearman ρ = 0.9966; Kendall τ = 0.9692).

Diagnostic Domain	Analysis Performed	Metric	Result
Leave-One-Drug-Out (LODO) Resampling	RF refitted leaving each drug out (25 iterations)	AUC	**0.776**
Train/Test Split (70/30)	Random hold-out evaluation	AUC	**0.583**
Variable Importance Stability	MeanDecreaseGini across LODO iterations	CV range (top predictors)	**0.13–0.32**
Reporting Volume Sensitivity	Model refitted excluding total_reports	AUC	**0.583**
Reporting Volume + Network Sensitivity	Model refitted excluding total_reports, mean_degree, mean_betweenness	AUC	**0.583**
Composite Weight Sensitivity	Alternative weights (0.50/0.20/0.20/0.10) vs original (0.60/0.15/0.15/0.10)	Spearman ρ	**0.9966**
		Kendall τ	**0.9692**
		Proportion changing decile	**23%**

Supplementary Table S9 — Range-based simulation of pregnancy exposure–liability ratios

Summary statistics from Monte Carlo simulations sampling pregnancy pharmacokinetic exposure within literature-reported minimum–maximum bounds. Ratios are dimensionless and represent exposure relative to ADMET-predicted potency thresholds (BSEP IC_{50} or CYP K_i, both in µM). Reported metrics include the median simulated ratio, 95% interval, and the probability that exposure exceeds unity (>1) or a high-margin threshold (>10).

Drug	Ratio definition	n draws	Median ratio	2.5%	97.5%	P(ratio > 1)	P(ratio > 10)
EFAVIRENZ	$Cavg_{(24)}$ / BSEP IC_{50}	4000	0.408	0.081	0.729	0.000	0.000
VALACICLOVIR	$Cavg_{(24)}$ / BSEP IC_{50}	2000	0.374	0.154	0.599	0.000	0.000
DOLUTEGRAVIR	$Cavg_{(24)}$ / BSEP IC_{50}	6000	0.183	0.122	0.290	0.000	0.000
DARUNAVIR	$Cavg_{(24)}$ / BSEP IC_{50}	6000	0.117	0.070	0.286	0.000	0.000
ATAZANAVIR	$Cavg_{(24)}$ / BSEP IC_{50}	6000	0.075	0.038	0.130	0.000	0.000
RILPIVIRINE	$Cavg_{(24)}$ / BSEP IC_{50}	6000	0.020	0.004	0.045	0.000	0.000

ABACAVIR	$Cavg_{(24)}$ / BSEP IC_{50}	4000	0.016	0.012	0.019	0.000	0.000
EMTRICITABINE	$Cavg_{(24)}$ / BSEP IC_{50}	4000	0.014	0.011	0.017	0.000	0.000
REMDESIVIR	$Cavg_{(24)}$ / BSEP IC_{50}	2000	0.003	0.003	0.003	0.000	0.000
OSELTAMIVIR	$Cavg_{(24)}$ / BSEP IC_{50}	2000	0.0003	0.0003	0.0003	0.000	0.000
EFAVIRENZ	$Cavg_{(24)}$ / CYP1A2 K_i	4000	13.8	2.75	24.7	1.000	0.664
RILPIVIRINE	$Cavg_{(24)}$ / CYP1A2 K_i	6000	0.424	0.090	0.938	0.003	0.000
EFAVIRENZ	$Cavg_{(24)}$ / CYP2C19 K_i	4000	2.35	0.467	4.20	0.838	0.000
DOLUTEGRAVIR	$Cavg_{(24)}$ / CYP2C9 K_i	6000	1.04	0.691	1.64	0.559	0.000
DARUNAVIR	$Cavg_{(24)}$ / CYP3A4 K_i	6000	10.4	6.16	25.3	1.000	0.540
ATAZANAVIR	$Cavg_{(24)}$ / CYP3A4 K_i	6000	2.65	1.36	4.60	1.000	0.000
EFAVIRENZ	Cmax / BSEP IC_{50}	4000	0.559	0.180	0.961	0.004	0.000
VALACICLOVIR	Cmax / BSEP IC_{50}	2000	1.24	0.612	1.83	0.688	0.000
EFAVIRENZ	Cmax / CYP3A4 K_i	4000	3.22	1.03	5.53	0.983	0.000
DARUNAVIR	Cmax / CYP3A4 K_i	6000	23.9	14.0	54.8	1.000	1.000

Supplementary methods

Supplementary Methods S1. Computational Workflow and Reproducibility Details

S1.1 Data Extraction, Structuring, and Unit of Analysis

Individual Case Safety Reports (ICSRs) were retrieved from the publicly accessible EudraVigilance portal for the predefined study period and processed to ensure internal consistency and reproducibility. All downstream analyses were derived from a single pregnancy ICSR population (N = 1,938 unique, deduplicated cases).

From this core pregnancy dataset, two analysis-ready representations were constructed:

1) Expanded reaction-level dataset (ICSR-level records)

Each MedDRA Preferred Term was retained as an individual row linked to its parent ICSR, with drug–ADR combinations represented explicitly. This reaction-level

structure was used for: phenotype clustering, ADR frequency analyses, and phenotype-level mechanistic enrichment.

2) One-row-per-case dataset (ICSR-level aggregation)

Records were collapsed using EU.Local.Number as the case identifier. Case-level variables were summarised as follows: seriousness indicators (maximum value per case), polypharmacy metrics (maximum number of active substances per case), and primary active drug (first non-missing suspect drug). This aggregated representation was used for sensitivity analyses and regression models to address non-independence.

All aggregation rules were implemented using dplyr::group_by() and summarise() to ensure reproducibility and transparent unit-of-analysis transitions.

S1.2 Primary Active Drug Assignment

For multi-drug regimens, a rule-based hierarchical classification was applied to assign a primary active drug. Drug classes were prioritised as follows:

Integrase strand transfer inhibitors (INSTI)

Protease inhibitors (PI)

Non-nucleoside reverse transcriptase inhibitors (NNRTI)

Nucleoside reverse transcriptase inhibitors (NRTI)

RNA-dependent RNA polymerase inhibitors (RdRp)

This ensured consistent linkage between pharmacovigilance data and drug-level mechanistic annotations.

S1.3 ADMET Feature Engineering

In Silico Annotation. SMILES structures for each primary active drug were processed using ADMET Predictor v11.

Generated features included:

Absorption parameters (e.g., permeability indices)

Distribution metrics (LogBB, Vd estimates)

Metabolic liabilities (CYP inhibition/substrate predictions)

Transporter interactions (BSEP, P-gp, BCRP)

Toxicity risk scores (TOX_Risk, MUT_Risk)

hERG inhibition predictions

Encoding

Continuous variables were retained as numeric values.

Binary inhibition/substrate flags were encoded as categorical variables.

For drug-level aggregation:

Continuous variables were averaged across associated ICSRs.

Binary variables were encoded using modal value (most frequent state).

Scaling

For embedding and composite scoring: Z-scores were computed across drugs using standard scaling (scale() in R).

S1.4 Polypharmacy and Network Feature Construction

Polypharmacy Metrics: suspect and concomitant medication lists were parsed into active ingredients.

Per ICSR, the following were computed:

Total active substances

Number of concomitant actives

Number of interacting actives

Indicator for ≥5 concomitant drugs

Drug-level polypharmacy metrics were calculated as means across ICSRs.

Co-Medication Network: A drug co-occurrence network was constructed based on within-ICSR co-prescription.

Nodes: active drugs

Edges: co-occurrence within the same ICSR

Network metrics computed using igraph:

Degree centrality

Betweenness centrality

Drug-level centrality measures were obtained by averaging node-level values.

S1.5 Disproportionality Analyses

For each drug–phenotype pair:

2×2 contingency tables were constructed

Reporting Odds Ratios (RORs) were computed

Fisher's exact tests were applied

Benjamini–Hochberg FDR correction was used

Disproportionality served as contextual annotation and was not used for predictive modelling.

S1.6 Random Forest Modelling

Objective: Estimate probability of high-burden drug classification.

Outcome Definition: High-severity drugs were defined as the upper quartile of composite ADR severity.

Model Specification

Algorithm: Random Forest

Implementation: ranger

Number of trees: 1000

Probability mode enabled

No class reweighting applied

Features included ADMET, polypharmacy, and network metrics

Performance Diagnostics

70/30 hold-out split (AUC reported)

Leave-one-drug-out (LODO) cross-validation

Out-of-bag (OOB) error

Bootstrap resampling (B = 500) for probability confidence intervals

Precision, recall, and F1 score for the minority class were computed at threshold 0.5.

S1.7 Composite Mechanistic Vulnerability Index (MVI)

The composite index was calculated as:

$$MVI=0.60\times \hat{P}_d+0.15\times Z_{\text{poly}}+0.15\times Z_{\text{network}}+0.10\times Z_{\text{mechanistic}}$$

Where:

$\hat{P}_d$ = RF-predicted high-risk probability

Z_{poly} = polypharmacy z-score

Z_"network" = network centrality z-score

Z_"mechanistic" = ADMET composite z-score

Alternative weight simulations (1000 random weight sets) were performed to test ranking stability.

Correlation matrices and variance inflation factors (VIF) were computed to assess multicollinearity.

S1.8 Dimensionality Reduction

PCA

Implementation: FactoMineR::PCA

Scaling: scale.unit = TRUE

Binary variables excluded

Loadings and eigenvalues reported

Leave-one-drug-out PCA sensitivity analyses were performed.

Embedding similarity was evaluated using Procrustes analysis (vegan::protest).

UMAP

Implementation: umap

Parameters: default unless otherwise specified

Used for visual pattern inspection only

S1.9 Exposure–Liability Simulations

Data Sources: Pregnancy-stage pharmacokinetic parameters (Cmax, AUC) were extracted from published literature.

Unit Harmonisation

Concentration values converted from µg/mL to µM using molecular weight

Average concentration: C_avg24=AUC_(0-24)/24

Sampling

For drugs with reported min–max bounds:

Uniform sampling across reported exposure range

Central value used when bounds unavailable

Monte Carlo simulation applied

Ratios Computed: Cmax / IC50 and Cavg / Ki

Outputs summarised as:

- Median
- 95% interval
- P(ratio > 1)
- P(ratio > 10)

These ratios were interpreted as plausibility margins, not predictive interaction models.

S1.10 Statistical Environment

All analyses were conducted in:

- R version 4.2.2 (Windows 10 x64)

Key packages:

- tidyverse
- igraph
- ranger
- pROC
- FactoMineR
- vegan
- sandwich
- lmtest
- pheatmap

No external machine learning automation frameworks were used.

The following table describes all engineered variables, system-level metrics, and mechanistic predictors used in the integrative analyses. Variables are grouped by analytical domain. Continuous variables were z-standardised prior to multivariate analyses. Directionality was harmonised such that higher values consistently indicate increased pharmacological complexity, mechanistic liability, or system-level vulnerability. ADMET predictor values represent in silico estimates and were used for mechanistic contextualisation rather than quantitative risk prediction.

Supplementary Table S10. Definitions of engineered variables, PCA inputs, and ADMET predictors used for mechanistic integration

Variable name (code)	Domain	Definition	Unit / Range	How computed	Interpretation
**Core engineered variables (ICSR- and drug-level)**					
poly_actives_total	Polypharmacy	Total number of distinct active drugs co-reported in an ICSR (suspect + concomitant)	Integer (≥0)	Count of unique active ingredients parsed from EV drug lists	Greater regimen complexity
mean_poly_actives_total	Polypharmacy	Mean polypharmacy burden per drug across its pregnancy-related ICSRs	Continuous	Mean(poly_actives_total) aggregated by drug	Higher background interaction burden
prop_ge5_concom	Polypharmacy	Proportion of a drug's reports with ≥5 concomitant active drugs	0–1	Mean(n_concom_actives ≥ 5) per drug	Higher likelihood of complex regimens
serious_prop	Outcome burden	Proportion of serious ICSRs for a given drug or phenotype	0–1	Mean(seriousness flag)	Higher severity burden
total_reports	Reporting burden	Number of pregnancy-related ICSRs associated with a drug	Integer	Count of unique EU.Local.Number per drug	Higher reporting exposure
log_total_reports	Reporting burden	Log-transformed total reports	Continuous	log10(total_reports + 1)	Stabilised reporting scale
n_clusters	Phenotypic breadth	Number of harmonised ADR phenotypes in which a	Integer (1–5)	Count of distinct cluster2 values per drug	Broader phenotype involvement

		drug appears			
dominant_cluster	Phenotype mapping	Harmonised ADR phenotype with the highest report count for a drug	Categorical	Mode of cluster2 per drug	Primary ADR phenotype
adr_composite	Empirical burden proxy	Composite proxy of reporting burden and phenotype breadth	Continuous	total_reports × n_clusters	Empirical burden (used for labeling only)
HighBurden	ML label	Binary indicator of high empirical burden	{0,1}	adr_composite ≥ 75th percentile	High reporting breadth
**Network topology variables**					
mean_degree	Network topology	Mean degree centrality in the drug co-reporting network	Continuous	Average node degree per drug	Greater co-medication connectivity
mean_betweenness	Network topology	Mean betweenness centrality in the drug co-reporting network	Continuous	Average betweenness per drug	Bridging / hub-like role
**PCA input variables (mechanistic–system-level embedding)**					
mean_poly_actives_total	Polypharmacy	Mean number of active drugs per ICSR	Continuous (z-score)	See above	Higher regimen complexity
mean_degree	Network	Mean network degree	Continuous (z-score)	See above	Higher system connectivity
mean_betweenness	Network	Mean network betweenness	Continuous (z-score)	See above	Greater topological influence
log_total_reports	Reporting	Log-scaled reporting volume	Continuous (z-score)	See above	Higher reporting exposure
n_clusters	Phenotypic breadth	Number of ADR	Integer (z-score)	See above	Broader ADR spectrum

		phenotypes per drug			
ADMET_Risk_mean	ADMETpredictor	Composite in silico ADMET risk score	Continuous (z-score)	Mean of ADMET risk predictors	Higher predicted liability
BSEP_IC50_mean	ADMETpredictor	Predicted BSEP inhibition potency	µM	Median IC50 per drug	Higher cholestatic liability
hERG_pIC50_mean	ADMETpredictor	Predicted hERG channel inhibition	pIC50	Median predicted value	Higher cardiac liability
CYP_HLM_CLint_mean	ADMETpredictor	Predicted intrinsic hepatic clearance	µL/min/mg	Median predicted clearance	Lower metabolic reserve
**ADMET predictors used in phenotype tests and scoring**					
CYP1A2_Ki	ADMETpredictor	Predicted CYP1A2 inhibition constant	µM	ADMET Predictor output	Higher enzyme inhibition
CYP2C9_Ki	ADMETpredictor	Predicted CYP2C9 inhibition constant	µM	ADMET Predictor output	Higher enzyme inhibition
CYP2C19_Ki	ADMETpredictor	Predicted CYP2C19 inhibition constant	µM	ADMET Predictor output	Higher enzyme inhibition
CYP2D6_Ki	ADMETpredictor	Predicted CYP2D6 inhibition constant	µM	ADMET Predictor output	Higher enzyme inhibition
CYP3A4_Ki	ADMETpredictor	Predicted CYP3A4 inhibition constant	µM	ADMET Predictor output	Higher enzyme inhibition
BSEP_IC50	ADMETpredictor	Predicted BSEP inhibitory concentration	µM	ADMET Predictor output	Higher transporter inhibition
MUT_Risk	ADMETpredictor	Composite mutagenicity risk score	Unitless	ADMET Predictor output	Higher genotoxic liability
MUTx_Risk	ADMETpredictor	Extended mutagenicity risk	Unitless	ADMET Predictor output	Higher genotoxic liability

S.MDCK	ADMETpredictor	Predicted MDCK cell permeability	nm/s	ADMET Predictor output	Higher membrane permeability

Pregnancy-specific pharmacokinetic (PK) and pharmacodynamic (PD) parameters were curated from the published literature to provide contextual interpretation and biological validation of pharmacovigilance and mechanistic findings. These parameters were not used as predictors in ADR clustering, disproportionality analyses, machine-learning models, or prioritisation score construction. Instead, PK/PD data were used to: 1) contextualise mechanistic vulnerability signals (e.g., exposure–liability relationships); 2) assess biological plausibility of phenotype-level enrichment patterns, and; 3) support exploratory exposure–liability ratio analyses.

Supplementary Table S11 - A. Pregnancy pharmacokinetic and pharmacodynamic sources by drug

Drug (Active Ingredient)	Dose (mg)	Regimen	Trimester / Period Covered	Reference
Efavirenz	600	Once daily	Third trimester; Postpartum	[29]
Dolutegravir	50	Once daily	Second and third trimesters; Postpartum	[30]
Oseltamivir	75	Twice daily	Not specified	[31]
Lopinavir	400; 600	Twice daily	Second trimester (400); Third trimester (600); Postpartum (400)	[32]
Ritonavir	100; 150	Twice daily	Second trimester (100); Third trimester (150); Postpartum (100)	[32]
Zidovudine	300	Twice daily	Postpartum (6–24 weeks)	[33]
Atazanavir (with cobicistat)	300	Once daily	Pregnancy; Postpartum	[34]
Nevirapine	200	Every 12 h	Antepartum; Postpartum	[35]
Bictegravir	50	Once daily	Second and third trimesters; Postpartum	[36]
Elvitegravir (with cobicistat)	150	Once daily	Second and third trimesters; Postpartum	[37]
Lamivudine	150	Twice daily	Postpartum	[33]
Abacavir	300	Twice daily	Third trimester	[38]
Darunavir (with cobicistat)	800	Once daily	Second and third trimesters; Postpartum	[39]
Emtricitabine	200	Once daily	Third trimester; Postpartum	[40]
Raltegravir	400	Twice daily	Second and third trimesters; Postpartum	[41]
Maraviroc	300	Twice daily	Third trimester; Postpartum	[42]
Rilpivirine	25	Once daily	Second and third trimesters; Postpartum	[43]
Tenofovir alafenamide	25	Once daily	Second and third trimesters; Postpartum	[44]
Remdesivir	100	Once daily	Third trimester	[45]
Valaciclovir	8000	Once daily	Third trimester	[46]

Supplementary Table S11 – B. Variables collected from each source

Variable	Domain	Description	Unit	Used in analysis
Cmax	PK	Maximum observed plasma concentration	µg/mL	Simulation & ratios
Cavg	PK	Average concentration over dosing interval	µg/mL	Simulation
AUC	PK	Area under concentration–time curve	µg·h/mL	Simulation & ratios
t½	PK	Elimination half-life	hours	Contextual
CL/F	PK	Apparent oral clearance	L/h	Contextual
Vd/F	PK	Apparent volume of distribution	L	Contextual
Viral load suppression	PD	Proportion of patients with VL <50 copies/mL	%	Interpretation
CD4+ count	PD	CD4+ lymphocyte count	cells/µL	Interpretation
